# *Mycobacterium tuberculosis* Binds Human Serum Amyloid A, and the Interaction Modulates the Colonization of Human Macrophages and the Transcriptional Response of the Pathogen

**DOI:** 10.3390/cells10051264

**Published:** 2021-05-20

**Authors:** Malwina Kawka, Anna Brzostek, Katarzyna Dzitko, Jakub Kryczka, Radosław Bednarek, Renata Płocińska, Przemysław Płociński, Dominik Strapagiel, Justyna Gatkowska, Jarosław Dziadek, Bożena Dziadek

**Affiliations:** 1 Department of Molecular Microbiology, Faculty of Biology and Environmental Protection, University of Lodz, 90-237 Lodz, Poland; malwina.kawka@biol.uni.lodz.pl (M.K.); katarzyna.dzitko@biol.uni.lodz.pl (K.D.); justyna.gatkowska@biol.uni.lodz.pl (J.G.); 2 Institute of Medical Biology, Polish Academy of Sciences, 93-232 Lodz, Poland; abrzostek@cbm.pan.pl (A.B.); jkryczka@cbm.pan.pl (J.K.); rplocinska@cbm.pan.pl (R.P.); pplocinski@cbm.pan.pl (P.P.); jdziadek@cbm.pan.pl (J.D.); 3 Department of Cytobiology and Proteomics, Medical University of Lodz, 92-215 Lodz, Poland; radoslaw.bednarek@umed.lodz.pl; 4 Biobank Lab, Department of Molecular Biophysics, Faculty of Biology and Environmental Protection, University of Lodz, 90-237 Lodz, Poland; dominik.strapagiel@biol.uni.lodz.pl

**Keywords:** *Mycobacterium tuberculosis*, serum amyloid A, human macrophages, host-pathogen interaction

## Abstract

As a very successful pathogen with outstanding adaptive properties, *Mycobacterium tuberculosis* (*Mtb*) has developed a plethora of sophisticated mechanisms to subvert host defenses and effectively enter and replicate in the harmful environment inside professional phagocytes, namely, macrophages. Here, we demonstrated the binding interaction of *Mtb* with a major human acute phase protein, namely, serum amyloid A (SAA1), and identified AtpA (Rv1308), ABC (Rv2477c), EspB (Rv3881c), TB 18.6 (Rv2140c), and ThiC (Rv0423c) membrane proteins as mycobacterial effectors responsible for the pathogen-host protein interplay. SAA1-opsonization of *Mtb* prior to the infection of human macrophages favored bacterial entry into target phagocytes accompanied by a substantial increase in the load of intracellularly multiplying and surviving bacteria. Furthermore, binding of human SAA1 by *Mtb* resulted in the up- or downregulation of the transcriptional response of tubercle bacilli. The most substantial changes were related to the increased expression level of the genes of two operons encoding mycobacterial transporter systems, namely, *mmpL5/mmpS5* (*rv0676c*), and *rv1217c*, *rv1218c*. Therefore, we postulate that during infection, *Mtb*-SAA1 binding promotes the infection of host macrophages by tubercle bacilli and modulates the functional response of the pathogen.

## 1. Introduction

Tuberculosis (TB) is a globally spread disease caused by the bacterial intracellular pathogen *Mycobacterium tuberculosis* (*Mtb*). According to the World Health Organization Global Tuberculosis Report 2020, in 2019 an estimated 10 million new cases of tuberculosis were diagnosed, and among these individuals, the alarming high mortality has reached 1.5 million deaths [1]. Although tuberculosis is considered a curable and preventable disease, it continues to pose a serious problem for modern medicine due to the startling increase in drug resistance of *Mtb* and rapid spread of the drug-resistant form of tuberculosis, which cannot be cured successfully with standard anti-tuberculosis treatment strategies [1,2]. This situation is also a significant challenge for world science. Moreover, we are still far from achieving a full understanding of the complex mechanisms used by tubercle bacilli to successfully gain access into, survive, and replicate within the hostile environment of targeted innate immune cells, namely, macrophages. Such knowledge is urgently needed for the search and selection of new therapeutic targets and the development of modern anti-tuberculosis drugs and vaccines. It is also vital to prevent tuberculosis from becoming incurable and to effectively control the spread of this disease.

*Mycobacterium tuberculosis* is one of the most successful intracellular pathogens that invade humans. This pathogen possesses outstanding adaptive properties, and over its long co-existence with human hosts, it has developed a plethora of sophisticated mechanisms to subvert host defenses and effectively enter and replicate in the harmful environment inside professional phagocytes, namely, macrophages. The pathogenic mechanisms of *Mtb* rely mainly on complex interactions with host innate immunity molecules and processes that are induced in response to infection. The invasion and survival strategies used by tubercle bacilli manipulate host innate immunity mechanisms and even hijack them from their original defense role to ensure the entry, dissemination, and persistence of the pathogen [3,4].

Infection with *Mtb* starts with the inhalation of airborne bacilli-bearing droplets released by individuals with active tuberculosis. After intrusion into the pulmonary alveoli, the invading *Mtb* initiates the host innate defense that begins with pattern recognition of the pathogen-associated molecular patterns (PAMPs) performed by a conserved group of host membrane-bound and soluble molecules, the so-called pattern recognition receptors (PRRs) [5,6]. Since the successful establishment of cellular invasion by pathogens requires adhesion to host cells, the interplay between *Mtb* cell wall components and host recognition molecules plays a crucial role not only in the mounting of an appropriate host first-line innate immune defense but also in tubercle bacilli entry into the target cells and intracellular multiplication and survival. Many studies have shown that within the alveolar microenvironment, tubercle bacilli interact with various membrane-bound PRRs expressed by macrophages and other immune cells, including Toll-like receptors, complement receptors, mannose receptors, CD14 receptors, Dectin-1, scavenger receptors, Fc*γ* receptors, and dendritic cell-specific intercellular adhesion molecule grabbing nonintegrin (DC-SIGN) [6]. In addition to the crosstalk between *Mtb* and cell surface host receptors, tubercle bacilli are able to interact with host soluble pattern recognition receptors, including C-type lectins (collectins), namely, mannose-binding lectin (MBL), pulmonary surfactant protein A (SP-A), pulmonary surfactant protein D (SP-D), and collectin-11 (CL-11); ficolins; complement component C3; and extracellular matrix proteins, such as fibronectin, heparin, and laminin [7].

The acute phase reaction (APR) is a systemic host response constituting a crucial element of the innate immune mechanism, and it is induced via local or systemic disturbances of macroorganism homeostasis. A wide variety of factors determine the development of APR, including microbial infections, tissue damage and pathology, and inflammatory processes of various etiologies. The clinical markers of APR are fever, tissue malnutrition and damage, vascular permeability changes and biosynthesis profile changes for more than 200 different proteins, so-called acute phase proteins (APPs), with the latter especially common for this type of response. The main sources of APPs are hepatocytes; however, these proteins are also produced by blood vessel wall cells and immunocompetent cells, namely, monocytes, lymphocytes, and alveolar macrophages. APPs synthesis is a consequence of a cellular response to stimulation by proinflammatory cytokines and chemokines produced and released by activated leukocytes, as well as epithelial cells, fibroblasts, and keratinocytes, with TNF-*α*, IL-1, and IL-6 playing key roles. The main criterion for the classification of APPs is at least a 25% change in their serum concentration during APR. Accordingly, the APPs were divided into two groups, namely, positive and negative, which are characterized by an increase or decrease in the serum protein level, respectively [8,9].

Among the many positive APPs, complement protein C3 and mannose binding lectin (MBL) have been shown to function as host innate PRR molecules that interact with the surface components of tubercle bacilli. Opsonization of *Mtb* by complement components and further interplay between opsonized bacteria and cell membrane complement “Trojan horse” receptors (CRs), namely, CR1, CR3, and CR4, are indicated as beneficial mechanisms facilitating the invasion of resident alveolar macrophages. This pathogen is able to hijack both classical and alternative pathways of complement activation to gain access to the complement fragments C3b and C3bi, respectively [10,11,12]. The mycobacterial 30 kDa heparin-binding hemagglutinin (Hbha) protein, and mannosylated lipoarabinomannan (ManLAM) and antigens of the Ag85 complex, namely, Ag85A and Ag85B, have been identified as *Mtb* ligands responsible for the interaction with a complement component C3 and MBL, respectively [13,14]. These antigens function as key virulence factors of tubercle bacilli and are involved in the successful pathogen entry into phagocytes, intracellular growth and survival, and manipulation of intracellular transport pathways and immune response mechanisms [14,15,16].

The main objective of the present paper was to explain the interplay of *Mtb* with human serum amyloid A, a highly conserved major positive acute-phase plasma protein synthesized in abundance (up to 1000-fold increases in the protein level in serum) by hepatocytes during the systemic APR response to environmentally harmful insults, such as infections, severe stress, and trauma. In addition to hepatocytes, in inflammatory tissues, the sources of SAA are macrophages, endothelial cells, smooth muscle cells and synoviocytes. There are four isoforms of SAA encoded by closely related genes, namely, SAA1, SAA2, SAA3, and SAA4. Of these four isoforms, SAA1 and SAA2 are acute phase proteins sharing 92% homology. Serum amyloid A serves as a major apolipoprotein of high-density lipoprotein (HDL), the predominant carrier of acute protein in the bloodstream. However, SAA can also associate with other lipoproteins, namely, low-density lipoprotein (LDL) and very low-density lipoprotein (VLDL). Serum amyloid A is a protein with pleiotropic functional activity. Both the high immunomodulatory potential and involvement in tissue repair processes make this protein necessary to maintain macroorganism homeostasis. The immunomodulatory activity of SAA is determined by its proinflammatory and anti-inflammatory properties. This acute phase protein opsonizes Gram-negative bacteria; presents chemotactic activity; stimulates cytokine synthesis by neutrophils, peripheral blood mononuclear cells, macrophages, and human mast cells; and promotes Th17-type immune response development and regulatory T-cell expansion. Thus, it serves as a regulator of innate and adaptive immunity. The immune properties of SAA are dependent on its interactions with cell surface receptors. There is no sole receptor dedicated to SAA. However, this protein can interact with Toll-like receptors (TLR2 and TLR4), class B scavenger receptors (SR-BI, LIMPII, CD36, CLA-1), receptors for advanced glycation end products (RAGE), and formyl peptide receptor-like 1 (FPRL1) [17,18].

The study described herein demonstrates the specific binding of SAA by *Mtb* and describes the effect of this interaction on the early stages of human macrophage infection by the pathogen. Furthermore, the effectors that determine the interplay of tubercle bacilli with SAA were identified and the functional response of *Mtb* to SAA binding at the transcriptome level was characterized.

## 2. Material and Methods

### 2.1. Mycobacterium tuberculosis Growth Conditions

*Mycobacterium tuberculosis* H37Rv strain was grown in 25 cm^2^ tissue culture flasks (Becton Dickinson, Burlington, NC, USA) in 10 mL of Middlebrook 7H9 broth (Difco, Baltimore, MD, USA) supplemented with BBL^TM^ Middlebrook 10% Oleic Albumin Dextrose Catalase (OADC) Enrichment (Difco, Baltimore, MD, USA) and 0.05% Tween 80 (Sigma Aldrich, St. Louis, MO, USA) until reaching an optical density of OD_600_ = 1.0 (6–7 days). For each experiment, freshly harvested bacterial cells were prepared by 20 min of centrifugation at room temperature and 4000× *g*. The pellet of mycobacteria was finally resuspended using the desired buffer or culture medium depending on the experimental procedure, and the optical density of the suspension was measured again to determine the number of bacterial cells per milliliter. According to preliminary experimental data employing the standard colony forming unit (CFU) method, an OD_600_ value of 1.0 corresponds to an *Mtb* suspension with a density of 1 × 10^8^ cells/mL.

### 2.2. Human Serum Amyloid A Binding by Mycobacterium tuberculosis

*Mycobacterium tuberculosis* H37Rv strain was cultured as described above, and once reaching the optical density of 1.0 at *λ* = 600 nm, the bacterial suspension volumes corresponding to the number of 2 × 10^8^ tubercle bacilli were collected by centrifugation and resuspended in 90 µL of prewarmed at 37 °C Iscove’s culture medium (Cytogen GmbH, Greven, Germany) supplemented with CaCl_2_ (Sigma Aldrich), to obtain a final concentration of 5 mM, and 0.1% bovine serum albumin (BSA), (Sigma Aldrich). To estimate the ability of live *Mtb* cells to bind human SAA, 10 µL of recombinant SAA1 (Sigma Aldrich) was added to experimental samples to reach final concentrations of 1 and 5 µg/mL. The mycobacterial cells prepared in 100 µL of the medium alone served as a negative control. Both experimental and control samples were then incubated for 90 min at 37 °C in a warm water bath with gentle stirring every 30 min. After the incubation period, the tubercle bacilli were spun down by centrifugation at room temperature and 16,000× *g* for 15 min, washed three times with 100 µL of Middlebrook 7H9 broth, and finally suspended in 500 µL of 0.5% formaldehyde (Sigma Aldrich) in phosphate buffered saline (PBS), (Cytogen GmbH, Greven, Germany) supplemented with 5 mM CaCl_2_. One hundred twenty microliters of the bacterial suspensions, which equals 4.8 × 10^7^ tubercle bacilli, were plated into the wells of 96-well polystyrene titration plates followed by overnight drying at 37 °C to fix the bacterial cells. The SAA1 binding experiments were performed in quadruplicate.

To detect recombinant SAA1 bound by live *Mtb* cells, a cellular enzyme-linked immunosorbent assay (cELISA) was performed. The wells of titration plates containing fixed mycobacteria were washed four times with a washing buffer, namely, PBS/0.05% Tween 20 (Sigma Aldrich), and then blocked using PBS enriched in 3% BSA (PBS/3% BSA). After overnight incubation at 4 °C, experimental and control samples were extensively washed six times (2 × 30 s, 4 × 5 min) with a washing buffer, and *Mtb*-bound SAA1 was detected using commercially available purified rabbit anti-human SAA1 primary antibodies (Sigma Aldrich) diluted 1:500 followed by six washes and the addition of horseradish peroxidase (HRP)-labeled goat polyclonal anti-rabbit IgG secondary antibodies (Jackson ImmunoResearch, West Grove, PA, USA) diluted 1:2000. Serological reactions with primary and secondary antibodies were carried out overnight at 4 °C. The resulting immune complexes were revealed by applying a mixture of ABTS (2,2′-azino-bis(3-ethylbenzothiazoline-6-sulfonic acid), (1 mg/mL), (Sigma Aldrich) as a chromogen and H_2_O_2_ (Sigma Aldrich) as an HRP substrate in a phosphate-citrate buffer, pH 4.5. The color enzymatic reaction was developed for 20 min at room temperature in the dark. The absorbance values of the experimental and control samples were measured at *λ* = 405 nm using a Multiscan EX (Thermo Fisher Scientific, Waltham, MA, USA) ELISA reader.

Optimal dilutions of primary and secondary antibodies used in the cELISA were determined in the preliminary titration experiments. The wells without *Mtb* cells served as a blank control for the cELISA test. All samples were run in triplicate.

### 2.3. Biotinylation of Recombinant SAA1

Recombinant human SAA1 was labeled with biotin using an EZ-Link^®^ Micro Sulfo-NHS-Biotinylation Kit (Thermo Fisher Scientific, Waltham, MA, USA) according to the manufacturer’s recommendation. The labeling efficiency of the human protein was confirmed with the application of the Western dot technique. Briefly, 5 µg of biotin-labeled SAA1 was loaded on a nitrocellulose membrane followed by 1 h of drying at room temperature and 1 h of blocking with a PBS/5% skim milk solution at room temperature with gentle shaking using a rocker shaker. After five washes (5 min each), the membrane was incubated in a 1:1000 diluted (PBS/0.05% Tween 20) solution of streptavidin-HRP polymer conjugate (Sigma Aldrich) at room temperature for 1 h. In the next step, the membrane was washed six times for 5 min each using a washing buffer, and the enzymatic reaction was developed using a chromogen, namely, 4-chloro-1-naphthol (Sigma Aldrich), at a concentration of 1 mg/mL, and H_2_O_2_ as a substrate, which were prepared together in a methanol (Sigma Aldrich)/PBS solution mixed at a *v/v* ratio of 1:4. Biotin-labeled human lactoferrin and PBS served as positive and negative controls of the test, respectively. Finally, the biotin-labeled SAA1 was aliquoted and stored at −20 °C.

### 2.4. Competitive Inhibition Assay

Freshly harvested 2 × 10^8^ *Mtb* cells were suspended in Iscove’s culture medium supplemented with CaCl_2_ (final concentration 5 mM) and 0.1% BSA, and then 5 µg of biotinylated recombinant SAA1 was added to each sample. To assess the specificity of the *Mtb*-SAA1 interaction, 1- (5 µg) and 3-fold (15 µg) excess unlabeled homologous protein, namely, human SAA1, was added to the appropriate experimental samples. Tubercle bacilli incubated with the labeled SAA1 exclusively were used as controls of the assay. The final volumes of the experimental and control samples were 100 µL. The competitive inhibition of binding of biotinylated SAA1 by the unlabeled homologue was carried out in a warm water bath at 37 °C for 2 h with gentle stirring every 30 min. Next, the tubercle bacilli were washed three times with Iscove’s medium employing centrifugation at room temperature and 4000× *g* for 20 min between each washing step. The bacterial cells were then resuspended in 500 µL of 0.5% formaldehyde in PBS with Ca^2+^ and Mg^2+^, and 150 µL of the experimental and control suspensions (6 × 10^7^ mycobacterial cells/well) were plated into the wells of 96-well titration plates followed by overnight drying at 37 °C. The competitive inhibition assay was performed in quadruplicate.

The level of binding inhibition of biotin-labeled SAA1 by tubercle bacilli in the presence of excess unlabeled homologous protein was determined with a cELISA. The fixed bacteria were washed four times with a PBS/0.05% Tween 20 buffer, and then, after blocking overnight with PBS/3% BSA at 4 °C, 200 µL of streptavidin-HRP polymer conjugate diluted 1:200 in PBS/0.05% Tween 20 (according to the manufacturer’s suggestion) was added to each well. The samples were incubated at room temperature for 1 h and washed four times. The color enzymatic-based reaction and absorbance measurement were performed as described above for the cELISA. All samples were run in triplicate.

### 2.5. Preparation of Mtb Whole-Cell Extract

Broken mycobacterial cell extract enriched in the membrane fraction of the proteins was prepared as previously described [19] according to the protocol of Heinz et al., [20] with the application of 1X PEN lysis buffer (100 mM Na_2_HPO_4_, 66.7 mM NaH_2_PO_4_, 150 mM NaCl, and 0.1 mM EDTA, pH 6.5), (Sigma Aldrich) and POP05 buffer (1X PEN buffer containing 0.5% (*m/v*) n-octylpolyoxyethylene, OPOE/(Sigma Aldrich). Initially, the freshly collected mycobacterial cells, which were resuspended in a cold PEN buffer, were disrupted using a BigB Lysing Matrix (MP Biomedicals, Irvine, CA, USA) and an ultrasound disintegrator (FastPrep-24, MP Biomedicals, Irvine, CA, USA) by the application of three 20 s cycles (6 m/s) performed at 3 min intervals on ice. Finally, after centrifugation at 16,000× *g* and 4 °C for 30 min, mycobacterial cell lysates were aliquoted and stored at 4 °C for further experiments. The protein concentration in the whole-cell extracts was determined using the Bradford Reagent (Sigma Aldrich) following the manufacturer’s instructions.

### 2.6. Binding of Human SAA1 by Native Mtb Proteins

The binding of human SAA1 by native *Mtb* proteins was evaluated using whole-cell mycobacterial proteins separated by one-dimensional (1D) and two-dimensional (2D) SDS-PAGE electrophoresis.

A one-dimensional electrophoretic technique was accomplished using commercial gradient (4–20%) Tris-Glycine polyacrylamide gels (Fermentas, Vilnius, Lithuania) and the Lane Marker sample buffer (Fermentas, Vilnius, Lithuania) containing 100 mM DTT as a reduction agent. Samples of 5–10 µg of protein were prepared in a buffer according to the manufacturer’s instructions, and then after a denaturation step at 37 °C for 20 min, they were subjected to electrophoresis at 100 V.

Prior to 2D SDS-PAGE electrophoresis, the mycobacterial protein extract was dialyzed against deionized AquaPurificata (Cytogen GmbH, Greven, Germany). The salt-free proteins were lyophilized and dissolved in a rehydration buffer (8 M urea, 2% CHAPS, 50 mM DTT, 1% ampholytes Bio-Lyte 3/10, 0.001% bromophenol blue), (Bio-Rad, Hercules, CA, USA) to a final concentration of 1 µg/mL. One hundred and twenty-five micrograms of the proteins was loaded into 7-cm nonlinear IPG ReadyStrips, pH 3–10 (Bio-Rad) using passive rehydration at 20 °C for 12 h. Subsequently, the 5-day isoelectric focusing (IEF) was performed at the same temperature using a Multiphor 211 (GE Healthcare, IL, USA) apparatus. After IEF, the gels were equilibrated with Tris-HCl buffers at pH 8.8 containing 6 M urea, 2% SDS, 2% DTT, and iodoacetamide, and then the proteins were resolved at 100 V by SDS-PAGE electrophoresis using 12% polyacrylamide gels.

The electrophoretically resolved proteins were then transferred to nitrocellulose membranes for 1.5 h at a voltage of 10 V using a Towbin buffer at pH 8.3 (25 mM Tris, 192 mM glycine, 0.5% SDS, 20% methanol). After overnight blocking with PBS/3% BSA at 4 °C, the washed membranes were subjected to 2 h of incubation with biotin-labeled human SAA1 at a final concentration of 10 µg/mL (1D electrophoresis) or 5 µg/mL (2D electrophoresis) in PBS with Ca^2+^ and Mg^2+^ enriched with 0.1% BSA at room temperature. To detect the protein-protein interaction of mycobacterial ligands with the human acute phase protein, the membranes were further incubated with streptavidin-HRP polymer conjugate diluted 1:1000 in PBS/0.05% Tween 20 for 1 h at room temperature. The colored reaction was developed with 4-chloro-1-naphthol at a final concentration of 1 mg/mL in PBS/methanol with the addition of 0.006% H_2_O_2_.

PageRuler^TM^Plus Prestained Protein Ladder (Fermentas, Vilnius, Lithuania) was used as a molecular weight marker.

### 2.7. Purification of Mtb Human SAA1-Binding Proteins

*Mycobacterium tuberculosis* proteins engaged in human serum amyloid A binding were isolated and purified with affinity chromatography and a Micro-Link^TM^ Protein Coupling Kit (Thermo Fisher Scientific, Waltham, MA, USA) according to the manufacturer’s protocol. In short, agarose resin (AminoLink Plus Coupling Resin) was coupled with 100 µg of recombinant human SAA1 prepared in 300 µL of Coupling Buffer with the addition of 2 µL of a 5 M sodium cyanoborohydride solution and 0.05% Tween 20. After 4 h of incubation at room temperature with adequate mixing, the remaining active sites of the washed resin were blocked with a Quenching Buffer, and, after the washing step, the resin was incubated overnight with 500 µg of mycobacterial whole-cell proteins at 4 °C with gentle end-over-end rotation. The possible nonspecific protein-protein interactions of the resin-coupled SAA1 with the mycobacterial ligands were eliminated by further 5-fold washing of the agarose resin with 0.5 M NaCl (Sigma Aldrich) containing 0.05% Tween 20 and then 3-fold washing with a Coupling Buffer. The human SAA1-bound *Mtb* proteins were next eluted by triple incubation (each of 10 min) of the chromatography resin with 100 µL of Elution Buffer at pH 2.8. The acidic eluates were then immediately neutralized by the addition of 5 µL of 1 M Tris (pH 9.0), and the eluted mycobacterial proteins were precipitated overnight at −20 °C using cold acetone at a final concentration of 25% (*v/v*). Finally, the precipitates were collected by centrifugation at 16,000× *g* and 4 °C, and, after drying at room temperature to remove any trace of acetone, they were resolved in 40 µL of cold PBS.

Analysis of the affinity chromatography-purified *Mtb* ligands binding of human SAA1 was carried out with one-dimensional standard SDS-PAGE electrophoresis under reducing conditions using gradient (4–20%) Precise Tris-Glycine Gels (Thermo Fisher Scientific, Waltham, MA, USA). Prior to electrophoresis, 5× sample buffer containing 4% (*w/v*) SDS and 5% (*v/v*) 2-mercaptoethanol was added to the protein samples, followed by heating at 37 °C for 20 min and resolution at a voltage of 100 V. The separated protein bands were visualized by staining with a commercial Imperial Protein Stain (Thermo Fisher Scientific Waltham, MA, USA), and their approximate molecular weights were determined based on the PageRuler^TM^Plus Prestained Protein Ladder, which was used as a molecular weight marker.

### 2.8. Identification of Mtb Proteins Binding Human SAA1

Sequencing of *Mtb* proteins engaged in the interaction with human SAA1 was performed at the Institute of Biochemistry and Biophysics Polish Academy of Sciences (Warsaw, Poland) using the liquid chromatography electrospray ionization tandem mass spectrometry (LC/ESI-MS/MS) analysis tool. Before the procedure, the affinity chromatography-purified human SAA1-binding mycobacterial proteins were separated by standard SDS-PAGE electrophoresis. The protein bands were then cut from the gel and sent for precise analysis. In addition, the identification procedure was also applied to mycobacterial proteins that presumably bind to human SAA1 and were selected via standard 1D and 2D electrophoretic techniques combined with Western blotting (Section 2.6).

Sequencing [21,22] and further identification of the mycobacterial proteins were accomplished according to the previously described procedures [19]. Valid protein identifications were identified by at least six peptides.

### 2.9. Cloning of the Mtb atpA, abc, espB, tb18.6, thiC, and ppiA Genes

Cloning of the *atpA, abc, espB, tb18.6, thiC,* and *ppiA* genes was performed according to a previously described procedure [19] using the total genomic DNA of the *Mtb* H37Rv strain as a template, appropriate primer pairs (Table S1), and pHis-Parallel1 expression vector. Restriction enzyme recognition sites were incorporated into the primer sequences (underlined sequences) to ensure subcloning into the expression vector. The resulting recombinant mycobacterial ligands were synthetized as fusion proteins with an additional 6-His Tag domain attached to their N-terminus [23]. The prepared constructs marked pHis-atpA, pHis-abc, pHis-espB, pHis-tb18.6, pHis-thiC or pHis-ppiA were finally introduced into *Escherichia coli* BL21(DE3) cells to induce biosynthesis of the recombinant forms of mycobacterial proteins, which were selected by affinity chromatography as presumably engaged in *Mtb*-human serum amyloid A interplay.

### 2.10. Expression and Purification of Recombinant rAtpA, rABC, rEspB, rTB18.6, rThiC, and rPpiA Proteins in Escherichia coli

Transformants of *E*. *coli* BL21(DE3) with introduced recombinant plasmid pHis-atpA, pHis-abc, pHis-espB, pHis-tb18.6, pHis-thiC or pHis-ppiA were used for overproduction of the corresponding recombinant *Mtb* protein. The recombinant proteins were purified by immobilized metal affinity chromatography using HisPur^TM^ Cobalt Spin Columns (Thermo Fisher Scientific, Waltham, MA, USA) or HisBind Ni-NTA Resin (Merck KGaA, Darmstadt, Germany).

To prepare highly purified recombinant mycobacterial proteins at a high final concentration the inclusion bodies were separated. Initially, the bacteria were cultured at 37 °C in a LB broth (Sigma Aldrich) supplemented with 100 µg/mL ampicillin as a selection antibiotic until the suspension reached an optical density of OD_600_ = 0.6. Efficient biosynthesis of the recombinant proteins was induced with 0.5 mM IPTG (Sigma Aldrich) for 3 h, and then the bacterial cells were harvested by centrifugation at 2880× *g* and 4 °C for 20 min. The obtained *E. coli* cell pellets were lysed using the BugBuster Protein Extraction Reagent (Merck KGaA, Darmstadt, Germany) with the addition of 1 mg/mL lysozyme (Sigma Aldrich), 25 U/mL benzonase nuclease (Sigma Aldrich), and 10 µg/mL phenylmethanesulfonyl (PMSF), (Sigma Aldrich), according to the protocol recommended by the manufacturer. Isolated inclusion bodies were dissolved in a Binding Buffer (Merck KGaA, Darmstadt, Germany) supplemented with 6 M urea (Sigma Aldrich) and 10 µg/mL PMSF. The insoluble residues were removed by centrifugation at 16,000× *g* and 4 °C for 30 min and subsequent filtration with 0.45 µm pore size microbiological filter units. The purification of the recombinant proteins from the obtained protein extracts was carried out under denaturing conditions using HisPur^TM^ Cobalt Spin Columns according to a previously described protocol [19] following the manufacturer’s instructions.

To prepare native (soluble) forms of recombinant rAtpA, rABC and rEspB, rTB18.6, and rThiC of *Mtb*, two different experimental strategies were employed.

Overproduction of native rAtpA and rABC by *E. coli* recombinant strains was induced with IPTG at a concentration of 0.4 mM at 25 °C for 4 h with constant shaking. These two proteins were purified from supernatants of the corresponding bacterial cell suspensions obtained by disruption of the bacteria with a Bioblock Scientific Ultrasonic Homogenizer (Labo Plus, Warsaw, Poland) using a His Bind Purification Kit (Merck KGaA, Darmstadt, Germany) containing His Bind affinity resin following the protocol adapted in our previous studies [19].

The biosynthesis of native rEspB, rTB18.6, and rThiC proteins by *E. coli* recombinants was induced in accordance with the above-presented protocol for purifying recombinant proteins from inclusion bodies. After lysis of the bacterial cells with the BugBuster Protein Extraction Reagent with addition of 1 mg/mL lysozyme, 25 U/mL benzonase nuclease, and 10 µg/mL PMSF, the insoluble debris was removed by centrifugation at 16,000× *g* and 4 °C for 30 min and subsequent filtration with 0.45 µm pore size microbiological filter units, and the protein preparations were subjected to further purification using 2.5 mL of 50% HisBind Ni-NTA Resin (Merck KGaA, Darmstadt, Germany) and His Bind Buffer Kit (Merck KGaA, Darmstadt, Germany). The settled resin that was loaded into plastic columns, was equilibrated with 3 volumes of sterile deionized water, 5 volumes of 1X Charge Buffer and 3 volumes of 1X Binding Buffer, and then the protein extract was passed through the affinity resin. Proteins unbound to the Ni-NTA resin were cleared away from the bed by extensive washing with 10 volumes of 1X Binding Buffer and 10 volumes of 1X Washing Buffer containing 20 mM (rThiC, rEspB) or 60 mM (rTB18.6) imidazole. The purified native mycobacterial proteins were eluted from the resin with 6 volumes of 1X Elute Buffer, and 1 mL fractions were collected and analyzed by SDS-PAGE electrophoresis with 12% polyacrylamide gels and staining with Imperial Protein Stain. Protein-rich eluates were further dialyzed against PBS at 4 °C using 10 kDa Slide-A-Lyzer Mini Dialysis Devices (Thermo Fisher Scientific, Waltham, MA, USA), and the concentration of each purified recombinant protein was quantified by the Bradford method using the Bradford Reagent.

The recombinant rAtpA, rABC, rEspB, rTB18.6, rThiC, and rPpi proteins were identified using Western blotting with the application of 0.2 µg/mL anti-His Tag mouse monoclonal antibodies (primary antibodies), (Merck KGaA, Darmstadt, Germany), 0.4 µg/mL HRP-conjugated anti-mouse IgG goat polyclonal antibodies (secondary antibodies), (Jackson ImmunoResearch, West Grove, PA, USA), and 4-chloro-1-naphthol as the chromogen. The recombinant protein bands were evaluated based on the theoretically calculated molecular masses, and PageRuler^TM^ Plus Prestained Protein Ladder or Spectra^TM^ Multicolor Broad Range Protein Ladder (Thermo Fisher Scientific, Waltham, MA, USA) was used as a molecular weight marker. The working concentrations of primary and secondary antibodies were established in the preliminary titration experiments.

### 2.11. Development of Rabbit Polyclonal Sera

New Zealand white laboratory rabbits were immunized with three doses (250 µg, 200 µg, and 200 µg) of rAtpA, rABC, rEspB, rTB18.6, rThiC or rPpi of *Mtb* protein, which were administered subcutaneously at 3-week intervals. Polyclonal sera were prepared from the blood of immunized animals, which was collected from anesthetized rabbits ten to twelve days after the last booster. The titers of recombinant protein-specific IgG antibodies and the optimal working dilutions of the developed rabbit serum immune system were estimated using indirect immunoenzymatic ELISA. Briefly, the wells of polystyrene MaxiSorp (Thermo Fisher Scientific, Waltham, MA, USA) plates were coated with 2 µg of mycobacterial recombinant protein as an antigen, and after blocking with PBS/10% FCS, serial dilutions (from 1:200 to 1:102,400) of the relevant rabbit serum (primary antibodies) were added in duplicate followed by 1 h of incubation at 37 °C. The complexes of primary antibodies with a specific recombinant antigen were detected using HRP-labeled anti-rabbit IgG goat polyclonal IgG immunoglobulins (secondary antibodies) and ABTS as the chromogen at a concentration of 1 mg/mL in a phosphate-citrate buffer at pH 4.5. The absorbance values were measured at *λ* = 405 nm using a MultiScan EX ELISA reader. The optimal dilution of secondary antibodies was determined in preliminary titration immunoenzymatic assays.

The New Zealand rabbits used for immunization experiments were raised under standard conventional conditions approved by the Polish Ministry of Science and Higher Education animal facility of the Institute of Microbiology, Biotechnology and Immunology, Faculty of Biology and Environmental Protection, University of Lodz. The applied procedures were approved and conducted according to guidelines provided by the appropriate Polish Local Ethics Commission for Experiments on Animals No. 9 in Lodz (Agreement No. 9/ŁB87/2018).

### 2.12. Binding of Human Serum Amyloid A by rAtpA, rABC, rEspB, rTB18.6, rThiC and rPpi Proteins

To confirm the ability of selected affinity chromatography identified by LC/ESI-MS/MS mass spectrometry *Mtb* proteins to bind human SAA1, the Western blotting technique was employed. In the first step, the mycobacterial proteins were separated under reducing conditions with standard SDS-PAGE electrophoresis using 12% polyacrylamide gels and then transferred to a nitrocellulose membrane under the abovementioned conditions (Section 2.6). Next, the remaining active sites of the nitrocellulose carrier were blocked overnight with a Western Blocker^TM^ Solution (Sigma Aldrich) at 4 °C, and after washing with PBS/0.05% Tween 20, the recombinant proteins were subjected to 2 h of interaction with human biotin-labeled SAA1 at a concentration of 10 µg/mL in PBS/0.05% Tween 20/0.1% BSA at room temperature. The protein-bound biotin-labeled SAA1 was detected by 1 h of incubation of the washed membranes with streptavidin-peroxidase polymer diluted 1:1000 in PBS/0.05% Tween 20, and finally, the colored reaction was developed with the chromogen 4-chloro-1-naphthol at a final concentration of 1 mg/mL in a PBS/methanol/H_2_O_2_ solution. PageRuler^TM^ Plus Prestained Protein Ladder or Precision Plus Protein^TM^ Dual Color Standard (Bio-Rad, Hercules, CA, USA) was used as the protein molecular weight markers.

### 2.13. Surface Plasmon Resonance

The interactions of the affinity chromatography-selected *Mtb* proteins with human serum amyloid A and their specificity were confirmed and further characterized by surface plasmon resonance (SPR) spectroscopy using a Biacore X system equipped with a CM5 Sensor Chip (Biacore AG, Uppsala, Sweden). Human SAA1 and BSA were diluted to a concentration of 100 µg/mL in a 10 mM sodium acetate buffer (pH 4.0) and then covalently attached to the surface of CM5 sensor chips using amine coupling chemistry (Biacore Amine Coupling Kit), (GE Healthcare, Uppsala, Sweden) according to the manufacturer’s instructions. A sensor chip with immobilized BSA was used as a control for assessing binding specificity and as a blank sample for subtraction of the bulk refractive index background. Binding of the selected mycobacterial recombinant native proteins, namely, AtpA (at concentrations of 2.5 µM, 5 µM, 10 µM, 20 µM), ABC (at concentrations of 2.5 µM, 5 µM, 10 µM, 20 µM), EspB (at concentrations of 202 nM, 403 nM, 1.008 µM, 2.017 µM, 4.034 µM), TB18.6 (at concentrations of 376 nM, 753 nM, 1.882 µM, 3.763 µM, 7.527 µM, 15.054 µM), and ThiC (at concentrations of 154 nM, 307 nM, 768 nM, 1.536 µM, 3.072 µM, 6.144 µM), to human SAA1 was measured over immobilized C1q at 22–25 °C in HBS-EP (0.01 M HEPES, 0.15 M NaCl, 3 mM EDTA, 0.005% *v/v* Surfactant P20, pH 7.4) as a running buffer with a flow rate of 5 µL/min (AtpA, ABC) or 20 µL/min (EspB, TB18.6, ThiC) and injection volumes of 30 µL (AtpA, ABC) or 35 µL (EspB, TB18.6, ThiC) followed by a 105 s period time of dissociation. Regeneration of the surfaces was achieved by injection of 20 µL 0.1 M glycine-HCl, pH 2.0 (60 s contact time). Sensorgrams were analyzed using the BIAevaluation 3.2 (AtpA, ABC) or BIAevaluation 4.1.1 (EspB, TB18.6, ThiC) software supplied by Biacore AB. The results are expressed in resonance units (RU), which are arbitrary units specific for a Biacore instrument (1000 RU corresponds to ~1 ng of bound protein/mm^2^). The association (k_a_) and dissociation (k_d_) rate constants were determined from individual association and dissociation phases, respectively, by global fitting of the data using the Langmuir binding model with a drifting baseline (assuming one-to-one interactions). Each dissociation constant (K_D_) was obtained by calculating the ratio of the dissociation and association rate constants: k_d_/k_a_ = K_A_ = K_D_^−1^. The range of SPR signals after binding of human SAA1 and BSA was 7850 RU and 2860 RU, respectively.

### 2.14. Preparation of Human Monocyte-Derived Macrophages (MDMs)

Human MDMs were differentiated from blood monocytes isolated from anonymized, commercially available buffy coats of healthy human blood donors (Regional Blood Donation Station, Lodz, Poland). Human blood monocytes were prepared with Histopaque-1077 (Sigma Aldrich) and 46% iso-osmotic Percoll (Sigma Aldrich) using a previously described double density gradient technique [24]. Briefly, after isolation of peripheral blood mononuclear cells with Histopaque-1077, blood monocytes were further purified from the cell suspension using 46% iso-osmotic Percoll according to the method of Danciger et al., [25] with slight modification. Finally, the human cells were suspended in Iscove’s medium with 4 mM L-glutamine and 25 mM HEPES (IMDM) supplemented with 0.005 mM 2-mercaptoethanol (Sigma Aldrich), 100 U/mL penicillin (Sigma Aldrich), 100 µg/mL streptomycin (Sigma Aldrich), and 10% human AB serum (Sigma Aldrich). The number and viability of the cells were determined using the Trypan blue (Bio-Rad) method. Differentiation of MDMs was performed within 7 days of culturing 5 × 10^5^ blood monocytes in 1 mL of culture medium containing 10 ng/mL macrophage colony-stimulating factor (M-CSF), (Thermo Fisher Scientific, Waltham, MA, USA) in 24-well tissue plates at 37 °C in a humidified atmosphere of 10% CO_2_/90% air. The purity of the obtained monocyte suspensions was established based on the morphological evaluation of cell preparations stained with Giemsa dye, under an optical microscope. The estimated purity of monocytes reached 80%.

### 2.15. Infection of Human MDMs with Mtb

To determine a presumable modulatory effect of human serum amyloid A binding by *Mtb* on pathogen entry into human macrophages, live 1 × 10^8^ *Mtb* cells were subjected to initial interactions with human SAA1 at final concentrations of 3 µg/mL and 15 µg/mL in an IMDM medium containing 0.1% BSA and 3 mM CaCl_2_ for 90 min at 37 °C (warm water bath) with gentle shaking every 30 min. The SAA1 concentrations used in the experiment corresponded to the average physiological and 5-fold increased serum levels of this acute protein in humans, respectively. *Mycobacterium tuberculosis* incubated in a medium without SAA1 served as a control. After the SAA1 binding step, the bacterial cells were washed with an IMDM medium alone and finally suspended in the same medium supplemented with 0.2% BSA and 0.005 mM 2-mercaptoethanol to a cell density 5 × 10^7^/mL.

Twenty-four hours before the *Mtb* infection, the cultures of human MDMs were thoroughly washed three times to remove any nonadherent cells and traces of antibiotics: Once with IMDM supplemented with 0.005 mM 2-mercaptoethanol, 100 U/mL penicillin, 100 µg/mL streptomycin, and 0.5% human AB serum; and twice with IMDM supplemented with 0.005 mM 2-mercaptoethanol and 0.5% human AB serum. Next, 1 mL of IMDM supplemented with 0.005 mM 2-mercaptoethanol and 10% human AB serum was added to each well with MDMs, and the cells were left resting overnight. Prior to infection, the medium was replaced with 900 µL of IMDM supplemented with 0.2% BSA and 0.005 mM 2-mercaptoethanol, and the macrophages were further infected with 100 µL of *Mtb* cells at an MOI of 1:10. After 2 h of incubation at 37 °C in a humidified atmosphere of 10% CO_2_/90% air, the extracellularly located tubercle bacilli were removed by extensive washing using IMDM supplemented with 0.005 mM 2-mercaptoethanol and 0.5% human AB serum, and the MDMs were subjected to further experimental evaluations. To determine the impact of SAA1 binding by *Mtb* on the early stages of human target cells infection or intracellular growth and the survival of the pathogen, MDMs were lysed with 0.1% SDS (Sigma Aldrich) immediately (day 0) or after 72 h (day 3) and 144 h (day 6) of further culturing. The appropriate dilutions of the macrophage lysates were plated onto a Middlebrook 7H10 agar (Difco, Baltimore, MD, USA) supplemented with 10% OADC enrichment, and after 21 days of growth, the number of colony forming units (CFU) was counted. To define the influence of SAA1 binding by *Mtb* on tubercle bacilli adherence to the target human cells, 2 h of infection was followed by elimination of cell-adhered bacteria by additional incubation of the washed MDMs with gentamicin at a concentration of 1 mg/mL, and after three washes with an IMDM medium supplemented with 0.005 mM 2-mercaptoethanol and 0.5% human AB serum, the human macrophages were immediately lysed (day 0). The optimal concentration of gentamicin providing efficient *Mtb* killing was determined in preliminary experiments.

The experiments were performed with macrophages from four independent healthy blood donors, with each run in triplicate.

### 2.16. RNA Isolation and Sequencing

The functional response of *Mtb* to the interplay with human serum amyloid A was evaluated at the transcriptome level for 2 × 10^8^ tubercle bacilli incubated with 15 µg/mL SAA1 in IMDM supplemented with 0.1% BSA and 3 mM CaCl_2_ for 3 h at 37 °C (warm water bath). The bacteria incubated with the medium alone served as a control. After the incubation time, the bacilli were centrifuged at 4000× *g* and room temperature for 20 min, and the bacterial cells were disrupted by bead beating with an ultrasound FastPrep-24 system using the TRIzol LS reagent (Thermo Fisher Scientific, Waltham, MA, USA) and two 45 s cycles (6 m/s) performed at 5 min intervals on ice as previously described [26]. Furthermore, DNA contamination of the RNA samples was removed using DNase I turbo (Thermo Fisher Scientific, Waltham, MA, USA) according to the manufacturer’s protocol. The integrity and quantity of RNA were examined by an Agilent 2100 BioAnalyzer following the manufacturer’s instructions (Agilent RNA 6000 Nano Kit), (Agilent Technologies, Santa Clara, CA, USA). The Illumina-compatible RNA/cDNA libraries were prepared as previously described [27]. AMPure XP magnetic beads (Becton Dickinson, Burlington, NC, USA) and Ribo-Zero rRNA Removal Kit (Illumina, San Diego, CA, USA) were used to purify RNA samples and remove ribosomal RNA, respectively. The sequencing libraries were prepared following the manufacturer’s instructions (KAPA Stranded RNA-Seq Kit), (KAPA Biosystems, Wilmington, MA, USA). Multiplex sequencing was performed using an Illumina True Seq v2 indexing system. The quantity and quality of libraries were inspected on an Agilent 2100 BioAnalyzer fitted with a DNA 1000 chip.

The RNA Seq libraries were sequenced using a NextSeq 500 System (Illumina and a NextSeq 500/550 Mid Output v2 Sequencing Kit (150 cycles), (Illumina), thus guaranteeing 5 to 10 million paired-end reads per sample. RNA isolation, library generation, and RNA sequencing were performed in three independent replicates.

### 2.17. RNA-Seq Data Analysis

RNA sequencing data analysis was processed with a series of software and bioinformatics scripts as previously described [27]. The sequencing adapters were removed with Cutadapt v.1.9.1 [28], and the reads were quality trimmed with a Sickle script, allowing a minimum quality of 30% and a minimal read length of 20 bp. The filtered reads were next aligned to the *Mtb* H37Rv genome (NC_018143.2) using the Bowtie 2 short read aligner [29]. Aligned data handling, conversion, and indexing were performed by the SAMtools [30] software suite. The differential expression was estimated using the online Degust RNA Seq analysis platform with default parameters (http://degust.erc.monash.edu) [31]. Gene expression was considered different if the false discovery rate value (FDER) was <0.05 and a log2-fold change was greater than an absolute value of 1 (changing two times or more).

### 2.18. Statistical Analysis

Data represented by the means ± SEM were analyzed using the Mann-Whitney U test. In some cases, the statistical evaluation of data from more than two experimental groups was performed via a one-way ANOVA. The differences were regarded as statistically significant at *p* < 0.05.

## 3. Results

### 3.1. Characterization of the Mtb Interaction with Human Serum Amyloid A

**Figure 1:**
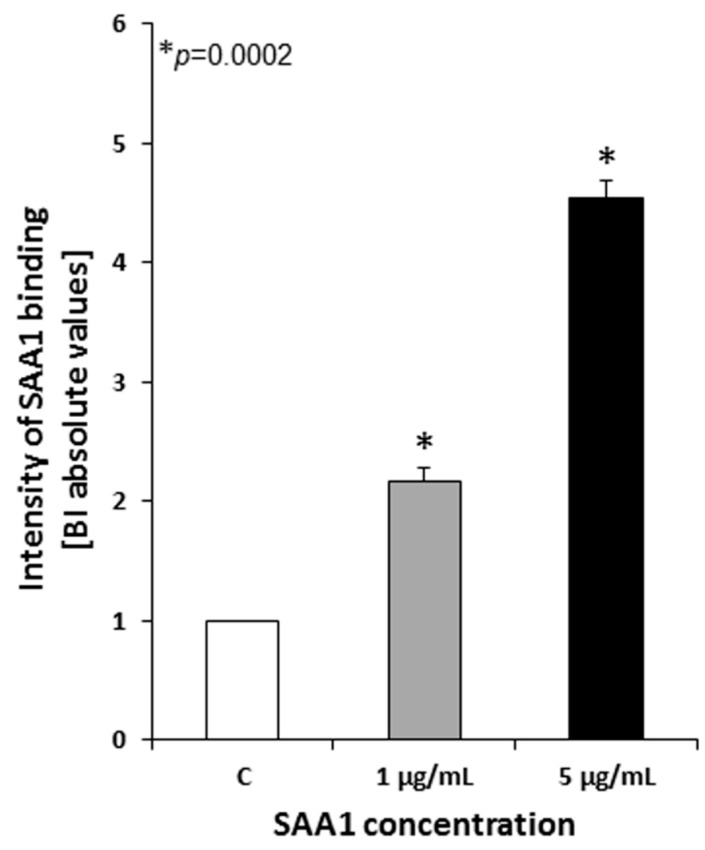
Binding of human SAA1 by live *Mycobacterium tuberculosis* cells. BI-binding intensity index; C-control tubercle bacilli incubated in a culture medium alone.

The ability of the pathogenic *Mtb* H37Rv strain to bind human SAA1 was evaluated using two different concentrations of human SAA1 (1 µg/mL and 5 µg/mL) and the cELISA method. The obtained data were calculated as a BI (binding intensity) index representing a quotient of the mean absorbance value of the test sample (*Mtb* cells incubated in the presence of SAA1) divided by the mean absorbance value of the corresponding control (*Mtb* cells incubated with the medium alone). Analysis of the cELISA results clearly demonstrated that tubercle bacilli essentially bind the human acute phase protein (Figure 1) (SAA1_1 µg/mL_: BI 2.17 ± 0.118, *p* = 0.0002; SAA1 _5 µg/mL_: BI 4.54 ± 0.151, *p* = 0.0002), and the amount of *Mtb*-bound SAA1 was directly proportional to its final concentration. The concentration-dependent intensity of *Mtb* interaction with SAA1 manifested as a statistically significant increase in the amount of the detected pathogen-bound protein in the test samples containing SAA1 at a concentration of 5 µg/mL compared to the test samples with SAA1 at a concentration of 1 µg/mL (*p* = 0.0002). 

**Figure 2:**
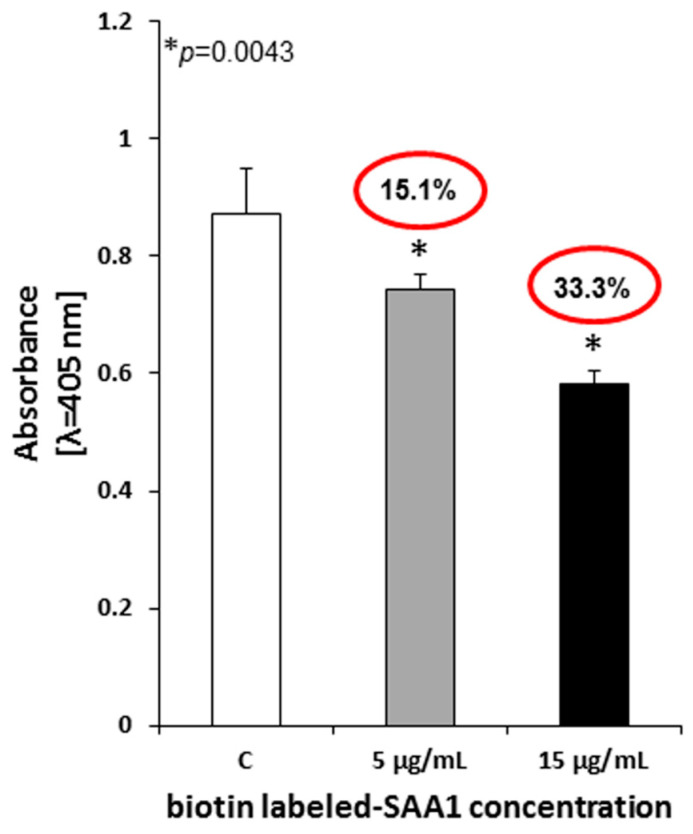
Inhibition of biotin-labeled human SAA1 binding by live *Mycobacterium tuberculosis* cells in the presence of 1-fold (5 µg/mL) and 3-fold (15 µg/mL) excess unlabeled homologous protein. C-control tubercle bacilli incubated with the addition of culture medium rather than unlabeled SAA1; the percent inhibition is marked in red circles.

To further characterize the nature of *Mtb* interplay with human serum amyloid A, the specificity of protein binding was determined using unlabeled (1-fold and 3-fold excess) and biotin-labeled (5 µg/mL) SAA1. Competitive inhibition experiments (Figure 2) revealed a significant decrease in the binding of biotin-labeled human SAA1 by *Mtb* in the presence of a 3-fold excess of unlabeled homologous protein compared to both controls (*Mtb* incubated with biotin-labeled SAA1 exclusively) (33.28%, *p* = 0.0043) and tubercle bacilli incubated with biotin-labeled SAA1 and a 1-fold excess of unlabeled acute phase protein (15.05%, *p* = 0.0043). Obviously, the above data confirmed the specific character of human SAA1 binding by live *Mtb* cells.

### 3.2. Selection of Mtb Ligands Involved in Human Serum Amyloid A Binding

**Figure 3:**
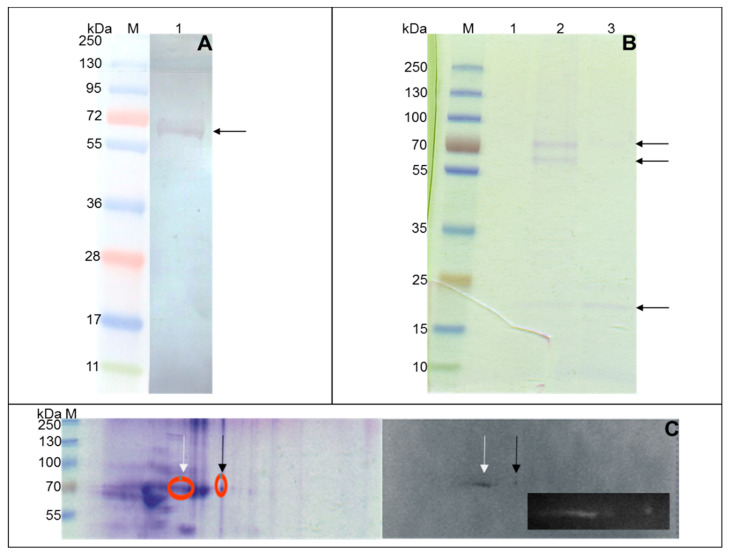
*Mycobacterium tuberculosis* ligands binding human SAA1 selected by the Western blot technique combined with 1D electrophoresis (**A**) and 2D electrophoresis (**C**), and by affinity chromatography (**B**). M-protein molecular weight standard; A-lane 1-binding of SAA1 by separated proteins of mycobacterial whole-cell lysate enriched in membrane protein fraction; B-lanes-1,2,3-mycobacterial ligands binding human SAA1 present in fractions 1, 2, 3 of affinity chromatography eluate, respectively; C-mycobacterial ligands binding human SAA1 are marked with arrows and red circles.

To accurately select *Mtb* membrane effector/effectors participating in the binding of human SAA1, a complex methodology was employed. Tubercle bacilli whole-cell extract enriched in the membrane fraction of the proteins was used as a source of the *Mtb* ligands potentially interacting with the human acute phase protein (Figure S1). The concentration of the proteins in this preparation was 1.7 mg/mL, as determined using the Bradford Reagent and method. Pathogen proteins were isolated using diverse laboratory techniques, namely, Western blotting combined with 1D or 2D electrophoresis and affinity chromatography with SAA1 coupled-beaded agarose resin. The SDS-PAGE profile of the pathogen ligands that bound the human acute protein revealed one (1D electrophoresis) (Figure 3A), two (2D electrophoresis) (Figure 3C) or three (affinity chromatography) (Figure 3B) intense protein bands of approximate molecular masses ranging from 20 kDa (affinity chromatography) to 60 kDa (affinity chromatography) and 70 kDa (Western blot and affinity chromatography). Additionally, the protein profile of the interaction of the tubercle bacilli proteome map with human SAA1 indicated the potential presence of different isoforms of the same *Mtb* protein or different pathogen proteins responsible for binding of the target human protein (Figure 3C).

### 3.3. Identification of Mtb Proteins Binding Human Serum Amyloid A

The selected *Mtb* proteins presumably interacting with human SAA1 and representing each band were identified further with liquid chromatography electrospray ionization tandem mass spectrometry (LC/ESI-MS/MS). Identification based on LC/ESI-MS/MS sequencing of at least six independent peptides (confidence level of at least 99%) from the same analyzed protein was required to include the candidate protein in the list of potential mycobacterial SAA1 ligands. As a final result, six candidate *Mtb* proteins, namely, AtpA (Rv1308; probable ATP synthase alpha chain AtpA), ABC transporter (Rv2477c; probable macrolide-transport ATP-binding protein ABC transporter), EspB (Rv3881c; secreted ESX-1 substrate protein B, EspB), TB 18.6 (Rv2140c; conserved protein TB18.6), ThiC (Rv0423c; probable thiamine biosynthesis protein ThiC), and PpiA (Rv0009; probable iron-regulated peptidyl-prolyl cis-trans isomerase A PpiA), were selected and subjected to further analysis. All identified mycobacterial proteins were detected in the membrane fraction of H37Rv *Mtb* cells [32,33] and therefore considered particularly interesting in the context of the pathogen-host interplay. Protein and peptide identification details are provided in Table S2 in the supplementary material related to this article.

To confirm the ability of the mass spectrometry-identified AtpA, ABC, EspB, TB18.6, ThiC, and PpiA *Mtb* proteins to bind human SAA1, recombinant forms of these proteins were prepared. SDS-PAGE and Western blotting with monoclonal mouse anti-6-His Tag IgG1 antibody analyses of rAtpA, rABC, rEspB, rTB18.6, rThiC, and rPpiA revealed that the target recombinant proteins were successfully expressed and purified by immobilized metal affinity chromatography (Figure S2). The SDS-PAGE-determined molecular masses of rAtpaA, rABC, rTB18.6, and rThiC of 62.8, 65.4, 22.3, and 63,4 kDa, respectively, were consistent with the values calculated from the amino acid sequences. However, the rEspB and rPpiA proteins migrated anomalously slower relative to the theoretically determined molecular masses of 51.2 and 22.8 kDa, respectively. Such atypical protein motility in SDS-PAGE electrophoresis, which is termed “gel shifting”, is common among membrane proteins and has been described in the literature [34,35]. It was suggested that the nature of this phenomenon could be related to altered protein-SDS loading arising from the abundance of hydrophobic residues in transmembrane regions of membrane proteins [34]. The purity of the recombinant protein preparations was evaluated using densitometry and FluorChem 8800 software (Alpha Innotech Corp., San Leandro, CA, USA), and the value was greater than 95%. The concentrations of rAtpA, rABC, rEspB, rTB18.6, rThiC, and rPpiA in the eluate fractions used for further experiments were 0.96, 1.01, 1.7, 1.2, 1.1, and 1.37 mg/mL, respectively, and they were determined by the Bradford method.

**Figure 4:**
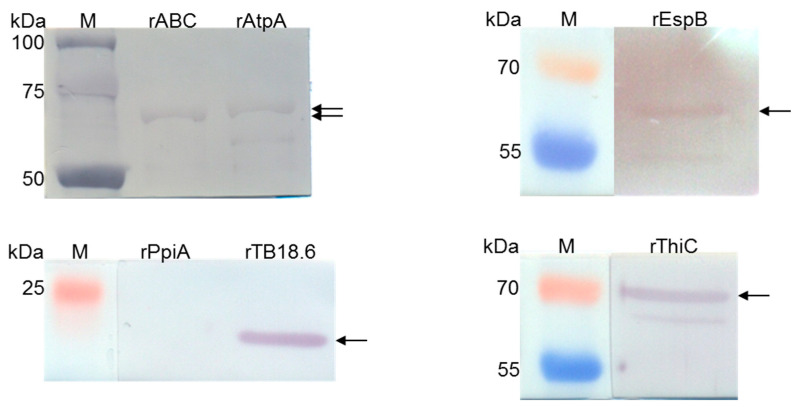
Western blot detection of the binding of biotin-labeled human SAA1 by recombinant *Mycobacterium tuberculosis* rABC, rAtpA, rEspB, rThiC, rTB18.6 and rPpiA proteins. M-protein molecular weight standards; the protein bands representing mycobacterial recombinant proteins with bound SAA1 are marked with arrows.

The capacity of *Mtb* recombinant proteins to interact with human SAA1 was evaluated with SDS-PAGE-separated proteins and biotin-labeled SAA1 using Western blotting. Immunodetection experiments showed that of the six initially identified SAA1 ligands of the pathogen, five proteins, namely, rAtpA, rABC, rEspB, rTB18.6, and rThiC, bound the human acute phase protein (Figure 4). A protein-SAA1 interaction was not noted for the rPpiA effector of *Mtb.*

**Figure 5:**
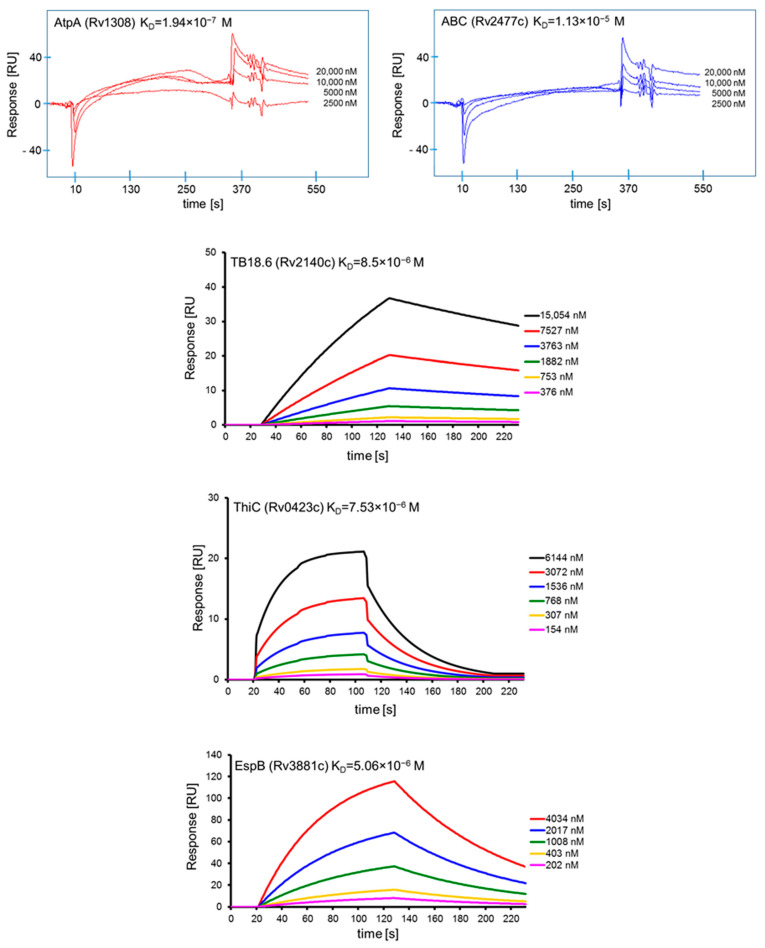
Surface plasmon resonance evaluation of the protein-protein binding interplay between recombinant *Mycobacterium tuberculosis* rAtpA, rABC, rTB18.6, rThiC, rEspB proteins (analytes) and human SAA1 immobilized onto a CM5 sensor chip. The presented kinetic data were calculated from at least three independent runs of each analyte concentration using Biacore AB BIAevaluation 3.2 (AtpA, ABC) or BIAevaluation 4.1.1 (TB18.6, ThiC, EspB) software.

The final verification and detailed analysis of the potential of five preselected *Mtb* proteins, namely, rAtpA, rABC, rEspB, rTB18.6, and rThiC, to bind human SAA1 were accomplished using the Surface Plasmon Resonance analytical tool. To confirm or exclude the previous observations, the human acute phase protein immobilized on the surface of the sensor chip was used as a ligand molecule and the soluble (native) recombinant mycobacterial proteins served as the analytes of the examined binding interactions, which were passed over the ligand via a continuous flow of the sample solution in separate runs. Differences in the refractive index between the running buffer and the injected sample were used to evaluate the bulk shift in the SPR response, and the k_a_ and k_d_ kinetic parameters were determined using the Biacore BIAevaluation softwares. Computer-designed SPR sensorgrams depict the kinetics of the examined *Mtb* protein-human SAA1 interactions and demonstrate the differences in the binding affinity depending on the pathogen ligand (Figure 5). The highest binding affinity was found for the interaction of mycobacterial rAtpA protein with SAA1, which was manifested by the lowest K_D_ value of 1.94 × 10^−7^ M noted for this protein-protein complex. Although less strong, the interaction of mycobacterial rEspB, rTB18.6, and rThiC was still significant at the affinity level, and the estimated K_D_ values were 5.06 × 10^−6^ M, 8.5 × 10^−6^ M, and 7.53 × 10^−6^ M, respectively. The weakest protein-protein binding interplay was found for the interaction between the *Mtb* rABC transporter and the human target acute protein. The calculated K_D_ value for this interplay was 1.13 × 10^−5^ M.

### 3.4. Interaction with Human Serum Amyloid A Favors Mtb Invasion of Human Macrophages

Since the interplay between *Mtb* and soluble PRRs, including acute phase proteins, is considered a substantial mechanism contributing to pathogen attachment and entry into target cells, it seems particularly valuable to determine the importance of human serum amyloid A binding by tubercle bacilli over the course of macrophage invasion. To assess the modulatory impact of SAA1 binding on the processes of entry, intracellular growth, and survival of *Mtb* in human macrophages, tubercle bacilli were subjected to interaction with the acute phase protein at the physiological concentration (3 µg/mL) and 5-fold higher concentration (15 µg/mL) prior to the in vitro infection of MDM cultures at an MOI of 10:1. The effect of SAA1 binding on pathogen entry into target phagocytes was estimated after 2 h of infection (day 0), and the potential changes in intracellular growth and survival were examined after 72 (day 3) and 144 (day 6) hours of infection.

**Figure 6:**
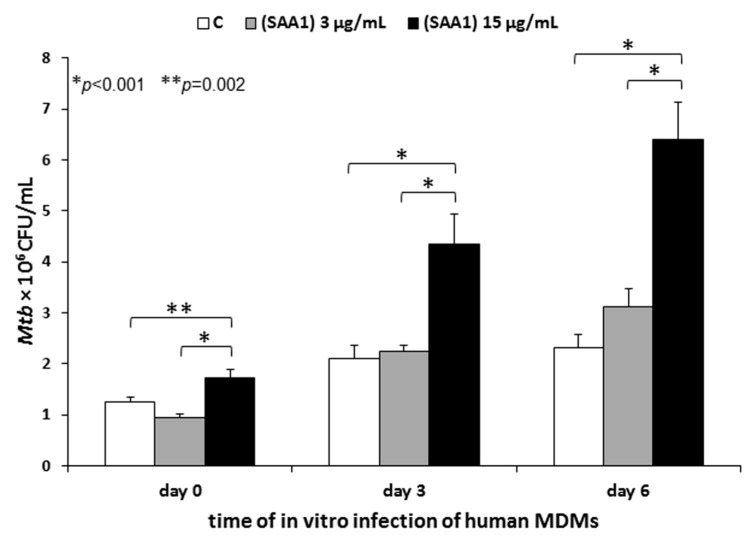
The binding interaction of *Mycobacterium tuberculosis* with human SAA1 at a concentration 5-fold higher (15 µg/mL) than the average physiological concentration prior to infection of human macrophages favors the colonization (day 0) of human macrophages and the number of intracellularly multiplying and surviving tubercle bacilli (day 3, day 6). C-control tubercle bacilli preincubated with a culture medium alone.

Analysis of the experimental data showed that the interaction of *Mtb* with SAA1 at a concentration of 3 µg/mL, which corresponded to the average individual physiological concentration of this acute phase protein, did not significantly modulate tubercle bacilli entry into human macrophages and did not affect intracellular growth and survival in the target phagocytes. However, valid changes in the examined processes were found for the bacteria preincubated with 5-fold higher, than the physiological, concentration of SAA1, which represented 16% of the detected average SAA concentration in the pleural fluids of tuberculosis patients [36]. The incubation of *Mtb* with SAA1 at a concentration of 15 µg/mL prior to the infection of human macrophages resulted in a significant increase in the number of intracellularly located bacterial cells, and the modulatory effect of SAA1 binding was significant compared to that of the control bacteria (*Mtb*/*Mtb*_SAA(15)_ 1.24 × 10^6^/1.73 × 10^6^ CFU/mL, *p* = 0.002), which were nonopsonized with the acute phase protein but also compared to the level of entry of tubercle bacilli that initially interacted with a physiological concentration of SAA1 (*Mtb*_SAA(3)_/*Mtb*_SAA(15)_ 0.94 × 10^6^/1.73 × 10^6^ CFU/mL, *p* < 0.001) (Figure 6). Moreover, opsonization of *Mtb* with a 5-fold higher concentration of SAA1 led to a significant enhancement in the number of intracellularly growing and surviving bacilli. Compared to the control bacteria, the number of live intracellularly growing SAA1-opsonized tubercle bacilli was two (*Mtb*/*Mtb*_SAA(15)_ 2.11 × 10^6^/4.35 × 10^6^ CFU/mL, *p* < 0.001) and almost three times (*Mtb*/*Mtb*_SAA(15)_ 2.32 × 10^6^/6.41 × 10^6^ CFU/mL, *p* < 0.001) elevated at days 3 and 6 of infection, respectively. Additionally, such as in the case of SAA1-promoted penetration of tubercle bacilli into human macrophages, acute phase protein concentration-dependent essential differences in intracellular growth and survival of *Mtb* were found (day 3: *Mtb*_SAA(3)_/*Mtb*_SAA(15)_ 2.24 × 10^6^/4.35 × 10^6^ CFU/mL, *p* < 0.001; day 6: *Mtb*_SAA(3)_/*Mtb*_SAA(15)_ 3.11 × 10^6^/6.41 × 10^6^ CFU/mL, *p* < 0.001).

In addition to the SAA1 concentration-dependent positive modulation of intracellular growth and survival, binding of SAA1 by *Mtb* resulted in a time-dependent promotion of these fundamental pathogenicity processes (day 0/day 3: *Mtb*_SAA(3)_ 0.94 × 10^6^/2.24 × 10^6^ CFU/mL, *p* < 0.001; *Mtb*_SAA(15)_ 1.73 × 10^6^/4.35 × 10^6^ CFU/mL, *p* < 0.001; day 3/day 6: *Mtb*_SAA(3)_ 2.24 × 10^6^/3.11 × 10^6^ CFU/mL, *p* = 0.016; *Mtb*_SAA(15)_ 4.35 × 10^6^/6.41 × 10^6^ CFU/mL, *p* = 0.009) (Figure 6). Contrary to tubercle bacilli preincubated with SAA1, the nonopsonized bacteria replicated significantly only during the first 3 days of infection of human macrophages (day 0/day 3: *Mtb* 1.24 × 10^6^/2.11 × 10^6^ CFU/mL, *p* = 0.003).

### 3.5. Impact of Human Serum Amyloid A Binding on the Functional Response of Mtb at the Transcriptome Level

**Figure 7:**
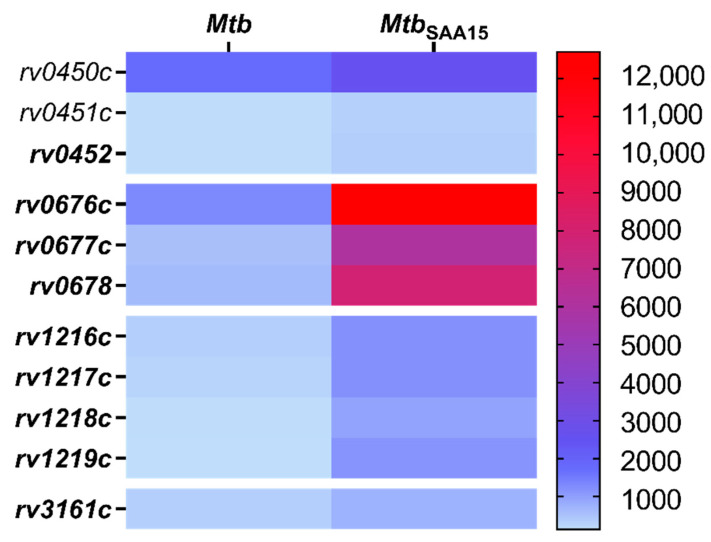
Heat map of differences in the number of transcripts identified by the RNAseq analysis of *Mycobacterium tuberculosis* interacting with a concentration of human SAA1 that is 5-fold higher than the average physiological concentration. *Mtb*-control tubercle bacilli incubated in medium alone; *Mtb*_SAA15_-tubercle bacilli incubated in a medium with human SAA1 at a final concentration of 15 µg/mL. The most substantial changes in the levels of transcripts are marked in bold.

To establish the influence of the interaction with human SAA1 on the functional response of *Mtb* at the transcriptome level, quantitative and qualitative analyses of RNA isolated from SAA1-osponized (15 µg/mL) and nonopsonized bacteria were performed using multiplex sequencing of Illumina technology. The results of a bioinformatics analysis of the obtained data showed significant differences in the levels of transcripts identified for tubercle bacilli interacting with SAA1. The noted changes were related to the increases in the expression levels of 11 genes (Figure 7). However, the most substantial changes were associated with genes of two different operons coding proteins of mycobacterial transporter systems, e.g., MmpL5/MmpS5 (Rv0676c/Rv0677c), permease (Rv1217c), and ATP-binding protein (Rv1218c) of the ABC-2 transporter system, and the gene for the mycobacterial dioxygenase (*rv3161c*). Additionally, the SAA1 binding-induced tendency for upregulation of transcripts expression levels was observed also for genes of another operon, namely, *rv0450c*, *rv0451c*, *rv0452*, encoding the mycobacterial MmpL4/MmpS4 transporter system. A detailed description of the gene products and changes in the levels of their transcripts compared to nonopsonized tubercle bacilli is depicted in Table 1 and Table S3.

## 4. Discussion

The prominent feature of *Mtb* pathogenicity is its ability to bind many host membrane and soluble molecules, including those serving as PRRs. [7,37]. Serum amyloid A, a key protein belonging to the group of positive APPs [8,9], is considered an interesting biomarker of lung disorders, including tuberculosis [38,39,40,41]. However, despite many years of research explaining the relationship between the level of SAA synthesis and the course of tuberculosis, our knowledge about the detailed role of this acute phase protein and the possible *Mtb*-SAA interaction in the modulation of the outcome of tubercle bacilli infection is still very poor.

In the present study, we found that *Mtb* specifically binds human SAA1 in a concentration-dependent manner and that this pathogen possesses membrane ligands, namely, AtpA (Rv1308; probable ATP synthase alpha chain AtpA), ABC (Rv2477c; probable macrolide-transport ATP-binding protein ABC transporter), EspB (Rv3881c; secreted ESX-1 substrate protein B, EspB), TB 18.6 (Rv2140c; conserved protein TB18.6), and ThiC (Rv0423c; probable thiamine biosynthesis protein ThiC), which are engaged in this interplay. Although our research proves for the first time that *Mtb* binds SAA1, this phenomenon should not be considered unexpected. First, previous researchers showed that tubercle bacilli bind human APPs, namely, C3 complement component [10,11,12,42] and MBL [14,16], and the mycobacterial ligands responsible for these interactions, namely, Hbha protein [13], and ManLAM and antigens of Ag85 complex (Ag85A, Ag85B) [14] have been identified. Moreover, similar to our observation of SAA1 binding (Figure S3), differences in the intensity of MBL binding between slow-growing *Mtb* and fast-growing *Mycobacterium* (*Mycolicibacterium*) *smegmatis* were observed [16]. Additionally, our competitive inhibition experiments excluded the specific nature of *M. smegmatis*-SAA1 interaction (Figure S4). Second, Hari-Dass et al. [43] described the concentration-dependent SAA1 binding by different Gram-negative bacteria, e.g., *E. coli*, *Salmonella typhimurium*, *Shigella flexneri*, *Pseudomonas aeruginosa*, *Vibrio cholera,* and *Klebsiella pneumoniae*. This phenomenon did not occur in Gram-positive bacteria, therefore, in the context of our experimental data, *Mtb* seems to be, so far, a unique Gram-positive bacterial pathogen that can bind human SAA1. Similar to differences in SAA1 binding by mycobacterial strains, the species-dependent specificity of SAA1 interaction with Gram-negative bacteria was also proved. Furthermore, the *E. coli* OmpA outer membrane protein, a member of the conserved family of OmpA/OprF proteins present in almost all Gram-negative bacteria, has been identified as a SAA1 binding ligand.

An obvious question that arises as a consequence of the proven ability of *Mtb* to specifically bind SAA1 is the importance of this interaction over the course of infection with this pathogen. To resolve this scientific puzzle, human MDMs were infected with tubercle bacilli opsonized with the physiological and 5-fold higher concentrations of the human acute phase protein. We found that the interaction of *Mtb* with the physiological concentration of SAA1 prior to the infection of human macrophages did not affect the pathogen invasion of the target phagocytes. However, a significant increase in the number of macrophage-entering bacteria was noted for the tubercle bacilli, which interacted with 5-fold higher, than the physiological concentration of SAA1. Furthermore, the rise in engulfed tubercle bacilli was accompanied by an elevated number of intracellularly growing and surviving bacteria. These data clearly suggest that during infection, the binding interaction of *Mtb* with human SAA1 could be a mechanism favoring colonization of human macrophages to ensure intracellular localization of the pathogen. Taking into account that alveolar macrophages possess features characteristic of both classically (phenotype M1) and alternatively (phenotype M2) activated macrophages [44] and possess suppressive properties due to the low expression of immunologically important receptors (e.g., TLR2) and costimulatory molecules (e.g., CD80, CD86) and limited ability to synthesize reactive oxygen radicals and weak bactericidal activity [45,46,47], the massive SAA1-promoted entry of tubercle bacilli into these cells might be an important mechanism used by tubercle bacilli to successfully multiplicate, persist, and disseminate in the infected host. Such a scenario is feasible due to the *Mtb* outstanding survival tactics that rely on the proven hijacking of alveolar macrophage diapedesis across the lung barrier for further spreading [48]. Furthermore, scientific reports [39,40,41] have documented a significantly increased concentration of SAA in patients with tuberculosis. Although the vast majority of these studies were focused on the comparative analysis of the serum SAA level, Samaha et al. showed elevated levels of this acute protein in both the sera and pleural fluids of *Mtb*-infected individuals [36]. The detected average concentration of SAA in the pleural fluids of tuberculosis patients was six times higher than that used in our studies and reached 93 µg/mL. Since as we showed, the amount of *Mtb*-bound SAA1 is protein concentration-dependent, it indicates that over the course of natural tubercle bacilli infection this interaction could be more intensive and result in an effective achievement of an intracellular niche inside alveolar macrophages. Discussing the result of our research, the importance of opsonization in the mounting of antimicrobial host defense cannot be forgotten. The binding of soluble humoral factors plays a vital role in recognizing invading pathogens by the dedicated receptors of cells of the innate immune system, including phagocytes, and enhances the effectiveness of phagocytosis, and in consequence the presentation of antigens, engagement of adaptive immunity, and final elimination of the pathogen. We cannot exclude that the binding interplay between *Mtb* and SAA1 is one of the host mechanisms facilitating the interaction of macrophages with tubercle bacilli, which the goal of successfully engulfing the pathogen and developing an appropriate complex immune response able to fight the infection. However, this process does not contradict the fact that opsonization by SAA1 may be exploited by *Mtb* for its own purposes. An important factor directing the outcome of the *Mtb*-SAA1 interaction could be the utilization of diverse SAA-specific receptors in the binding of opsonized tubercle bacilli, namely, Toll-like receptors TLR2 and TLR4, N-formyl peptide receptor 2 (FPR2) or class B scavenger receptor SR-B2 (CD36). Since *Mtb* could influence the regulation of an array of host signal transduction pathways, including modulation of macrophage functions, the type of PRRs used by tubercle bacilli might be crucial for pathogen entry into target cells and intracellular survival or induction of efficient host first-line innate immune defense [49]. For other human acute phase proteins, namely, complement C3 components, tubercle bacilli were shown to utilize these opsonins and surface “Trojan horse” complement receptors of the phagocytes, namely, CR1, CR2, and CR3, to gain access to the target cells. However, the most substantial mechanism is the interaction of C3bi-opsonized mycobacteria with CR3, which leads to the inhibition of respiratory burst and the inflammatory response of macrophages [12].

In addition to modulatory effects on the invasion of human macrophages, we also found that the binding of SAA1 affects the transcriptional response of *Mtb*. The noted changes manifested as an increase in levels of transcripts for genes located within three operons that code transport proteins, namely MmpL4/MmpS4 (Rv0450c/Rv00451c/Rv0452), MmpL5/MmpS5 (Rv0676c/Rv0677c/Rv0678), and ABC-2 (Rv1217c/Rv1218c/Rv1219c), or the gene that codes dioxygenase. However, the most substantial differences were observed in the expression levels of the *mmpL5*/*mmpS5* and *rv1217c*/*rv1218c*/*rv1219c* genes, and this positive shift was potentially related to the elevated target protein synthesis and could result in improvement of *Mtb* pathogenicity. Mycobacterial MmpL transmembrane proteins are members of the resistance-nodulation-cell division (RND) subfamily of bacterial multidrug efflux pumps involved in the active transport of a wide range of substrates, including fatty acids, powered by a proton-substrate antiport [50]. Wells et al. proved that the fundamental functional activity of the MmpL5/MmmS5 protein complex is related to the export of siderophores, namely, mycobactin and carboxymycobactin, by their translocation across the inner membrane into the periplasmic space. Biosynthesis of mycobactin and carboxymycobactin is induced under low-iron conditions, and *Mtb* employs its siderophores to capture iron from host iron stores [51]. Since iron is an essential nutrient required for intracellular growth and *Mtb* persistence and access to iron is restricted in alveolar macrophages by the NrampI macrophage protein (natural resistance-associated macrophage protein) [52,53], the elevated expression of *mmpL5*/*mmpS5* could be a key strategy of tubercle bacilli to overcome this host defense mechanism. Mycobactin and carboxymycobactin increase the availability to the iron of phagosomal compartment by almost 20-fold [54]. Therefore, the successful transport of these siderophores might be of importance in maintaining *Mtb* virulence. Deletion of *mmpL* genes, including *mmpL5*, drastically decreased the synthesis and secretion of siderophores in *Mtb* and diminished the ability of tubercle bacilli to survive in mouse lungs by 100-fold [51]. In addition to mycobactin and carboxymycobactin export, the MmpL5/MmpS5 protein complex is required for siderophore recycling, and this mechanism is considered enables *Mtb* to acquire iron at low metabolic costs during infection [55].

ATP binding cassette (ABC) transporters constitute a large group of multisubunit permeases involved in relative substrate-specific transport of various molecules across biological membranes. *Mycobacterium tuberculosis* ABC transport proteins are categorized into nine different subfamilies [56]. The product of the *rv1218c* gene of *Mtb*, whose expression level was substantially increased after SAA1 binding, is supposed to provide tubercle bacilli drug resistance by mediating the efflux of a wide range of chemicals and antibiotics, including biarylpiperazines, pyridines, pyrroles, bisanilinopyrimidines, and novobiocins [57]. Furthermore, overexpression of ABC transporters, including Rv1217c and Rv1218c, was proven to significantly contribute to the resistance of *Mtb* to anti-tuberculosis drugs, namely, rifampicin and isoniazid [58]. In addition, a correlation between *Mtb* drug resistance and increased ABC efflux pumps expression was also reported for other members of this protein superfamily. Choudhuri et al. found that overexpression of ABC proteins encoded by *drrA* and *drrB* genes conferred resistance to various clinically relevant and structurally unrelated antibiotics, namely, tetracycline, erythromycin, ethambutol, norfloxacin, streptomycin, and chloramphenicol [59]. In the context of the above data, the *Mtb*/SAA1-binding driven increase in the levels of transcripts of the *rv1217c* and *rv1218c* genes seems to be one of the prominent mechanisms favoring *Mtb* virulence and survival in infected hosts.

Five *Mtb* membrane protein ligands, namely, AtpA, ABC, EspB, ThiC, and TB18.6, have been confirmed to confer the pathogen with the ability to bind human SAA1. The binding of a single host protein by various *Mtb* effectors was also noted for interactions with MBL (ManLAM, Ag85A, Ag85B) [14], surfactant protein A (ManLAM, lipomannan, Apa glycoprotien) [60,61], and extracellular matrix components, namely, fibronectin (Ag85A, Ag85B, glycoprotein Apa, GlcB adhesin) [62,63,64] and laminin (GlcB adhesion, ESAT-6 secretory adhesion) [64,65]. Moreover, in our previous studies on the interaction of *Mtb* with the host chemotactic cytokine network, we found that tubercle bacilli are able to bind human IL-8 using AtsG, GlmU, and SahH membrane proteins [19]. Although the outcome of these interactions is still not fully understood, they provide more efficient pathogen colonization of host target cells. Among the five proteins participating in the interplay of *Mtb* with human SAA1, the highest binding affinity was observed for the AtpA ligand, which constitutes the alpha chain of mycobacterial ATP synthase that is involved in the synthesis ATP from ADP in the presence of a proton gradient across the membrane. This membrane protein, similar to the other four proteins, was not described previously as a mycobacterial ligand interacting with host soluble or membrane components. However, Huo et al. adapted a computational tool to predict protein-protein interplay and indicated the potential of regulatory subunits of ATP synthase, including AtpA protein, for interaction with host targets [66]. Moreover, under the dormancy state characteristic for latent *Mtb* infection, the bacteria demonstrate an altered physiological status manifested as reduced replication and metabolism accompanied by downregulation of genes encoding also ATP synthase [67]. These data are consistent with observations by Rao et al., who found that the intracellular concentration of ATP in hypoxic nonreplicating tubercle bacilli is significantly (five to six times) lower compared to that of the control bacteria multiplying under aerobic conditions [68]. In addition, a proteomic comparative analysis of *Mtb* clinical isolates resistant to amikacin and kanamycin indicating indicated that the AtpA subunit is a promising target for new anti-tuberculosis drugs [69]. However, further research is needed to fully understand both the involvement of AtpA and its interaction with SAA1 in *Mtb* pathogenicity.

In conclusion, the present study proves the binding of human SAA1 by *Mtb* and demonstrates that upregulated synthesis of this acute protein during tubercle bacilli infection promotes mycobacterial entry into target human macrophages along with an elevated number of intracellularly growing and surviving bacteria. The binding interplay between *Mtb* and SAA1 modulates the transcriptional response of the pathogen, thus leading to the substantial expression of genes encoding proteins related to pathogen virulence. Additionally, tubercle bacilli possess five putative membrane ligands engaged in SAA1 binding with various affinities. The identified interaction between the pathogen and SAA1 needs to be further studied to clearly explain possible positive and negative outcomes for both the infected individual and invading tubercle bacilli.

## Figures and Tables

**Table 1:** Functional response of *Mycobacterium tuberculosis* to the interaction with human SAA1 at the transcriptome level.

Gene		Changes in Expression Level	*p*	Gene Product
*rv0450c*	*mmpL4*	↑0.401	3.98 × 10^−6^	conserved transmembrane transport protein MmpL4
*rv0451c*	*mmpS4*	↑0.781	2.90 × 10^−5^	conserved membrane protein MmpS4
***rv0452***	**-**	**↑1.039**	2.75 × 10^−8^	transcriptional regulatory protein
***rv0676c***	***mmpL5***	**↑2.997**	1.94 × 10^−19^	conserved transmembrane transport protein MmpL5
***rv0677c***	***mmpS5***	**↑3.141**	4.91 × 10^−18^	conserved membrane protein MmpS5
***rv0678***	**-**	**↑3.298**	1.02 × 10^−17^	transcriptional regulatory protein
***rv1216c***	**-**	**↑1.621**	2.73 × 10^−14^	conserved integral membrane protein
***rv1217c***	**-**	**↑1.891**	2.97 × 10^−13^	tetronasin-transport integral membrane protein ABC transporter
***rv1218c***	**-**	**↑2.573**	6.77 × 10^−14^	tetronasin-transport ATP-binding protein ABC transporter
***rv1219c***	**-**	**↑2.679**	2.36 × 10^−13^	transcriptional regulatory protein
*rv3161c*	-	↑0.947	6.00 × 10^−8^	dioxygenase

↑ Increase of expression, the most substantial changes in levels of transcripts are marked in bold.

## Data Availability

The data presented in this study are available online (see supplementary files) or upon request from the corresponding author.

## Supplementary Materials

The following are available online at https://www.mdpi.com/article/10.3390/cells10051264/s1, Figure S1: SDS-PAGE analysis of the quality of *Mycobacterium tuberculosis* whole-cell protein extract used for isolation of the ligands binding human SAA1; Figure S2: Western blot analysis of recombinant *Mycobacterium tuberculosis* rAtpA, rABC, rThiC, rEspB, rPpiA, and rTB18.6 proteins developed in *E. coli* with anti-His Tag mouse monoclonal IgG1 antibodies; Figure S3: Intensity of human SAA1 binding by live Mycobacterium tuberculosis (Mtb) and Myco-bacterium smegmatis (Msmeg) cells. BI-binding intensity index; C-control Mtb and Msmeg cells in-cubated in culture medium alone; Figure S4: Inhibition of biotin-labeled human SAA1 binding by live Mycobacterium smegmatis cells in the presence of 1-fold (5 µg/mL) and 3-fold (15 µg/mL) excess of unlabeled homologous protein. C-control bacilli incubated with the addition of culture medium instead of unlabeled SAA1; the percent of inhibition is marked in red circle; Table S1: Primer sequences used for PCR amplification of the gene sequences; Table S2: *Mycobacterium tuberculosis* SAA1 binding protein and peptide list identified by LC/ESI-MS/MS; Table S3: RNAseq data base.
