# Supplementary material for: Mycobacterium tuberculosis Binds Human Serum Amyloid A, and the Interaction Modulates the Colonization of Human Macrophages and the Transcriptional Response of the Pathogen
Source: Cells. 2021 May 20;10(5):1264. doi: 10.3390/cells10051264 (PMC8160739; doi:10.3390/cells10051264)
Supplement: Supplementary file 1 [file cells-10-01264-s001.zip › cells-1193738-supplementary.docx]

*Mycobacterium tuberculosis* binds human serum amyloid A, and the interaction modulates the colonization of human macrophages and the transcriptional response of the pathogen.

Malwina Kawka, Anna Brzostek, Katarzyna Dzitko, Jakub Kryczka, Radosław Bednarek, Renata Płocińska, Przemysław Płociński, Dominik Strapagiel, Justyna Gatkowska, Jarosław Dziadek and Bożena Dziadek

Supplementary data:

**Figure S1.** SDS-PAGE analysis of the quality of *Mycobacterium tuberculosis* whole-cell protein extract used for isolation of the ligands binding human SAA1.

**Figure S2.** Western blot analysis of recombinant *Mycobacterium tuberculosis* rAtpA, rABC, rThiC, rEspB, rPpiA and rTB18.6 proteins developed in *E. coli* with anti-His Tag mouse monoclonal IgG1 antibodies.

**Figure S3**. Intensity of human SAA1 binding by live *Mycobacterium tuberculosis* (*Mtb*) and *Mycobacterium smegmatis* (*Msmeg*) cells. BI-binding intensity index; C-control *Mtb* and *Msmeg* cells incubated in culture medium alone.

**Figure S4.** Inhibition of biotin-labeled human SAA1 binding by live *Mycobacterium smegmatis* cells in the presence of 1-fold (5 µg/ml) and 3-fold (15 µg/ml) excess of unlabeled homologous protein. C-control bacilli incubated with the addition of culture medium instead of unlabeled SAA1; the percent of inhibition is marked in red circle.

**Table S1.** Primer sequences used for PCR amplification of the gene sequences.

**Table S2.** *Mycobacterium tuberculosis* SAA1 binding protein and peptide list identified by LC/ESI-MS/MS.

**Table S3.** RNAseq data base (see Excel file)


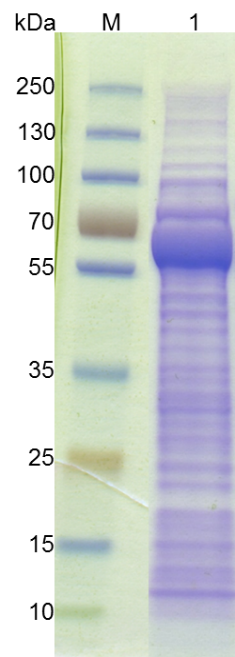


**Figure S1.** SDS-PAGE analysis of the quality of *Mycobacterium tuberculosis* whole-cell protein extract used for isolation of the ligands binding human SAA1.


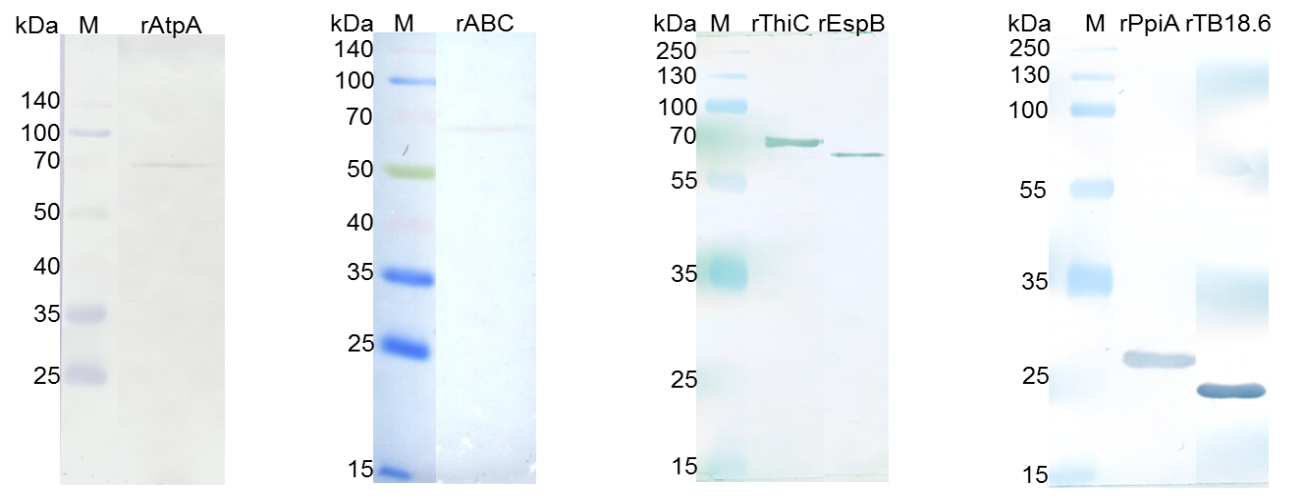


**Figure S2.** Western blot analysis of recombinant *Mycobacterium tuberculosis* rAtpA, rABC, rThiC, rEspB, rPpiA and rTB18.6 proteins developed in *E. coli* with anti-His Tag mouse monoclonal IgG1 antibodies.


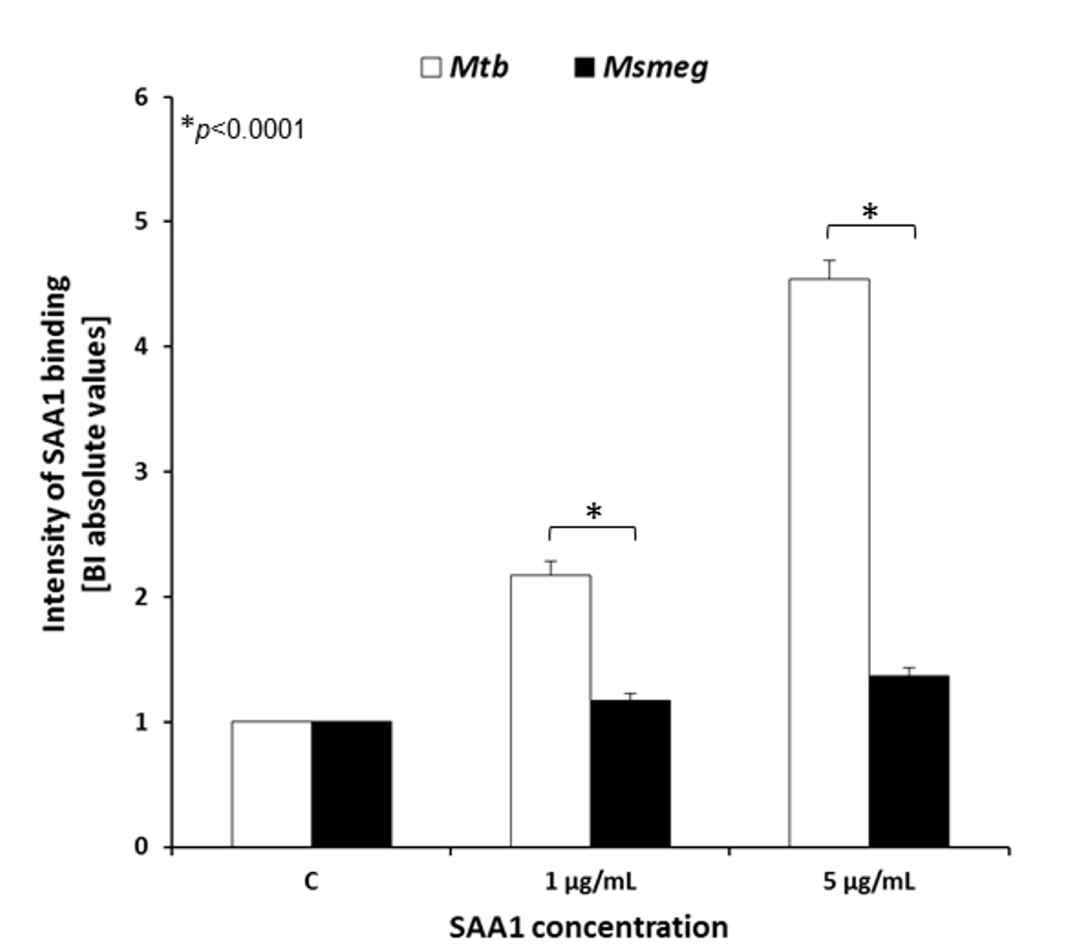


**Figure S3.** Intensity of human SAA1 binding by live *Mycobacterium tuberculosis* (*Mtb*) and *Mycobacterium smegmatis* (*Msmeg*) cells. BI-binding intensity index; C-control *Mtb* and *Msmeg* cells incubated in culture medium alone.


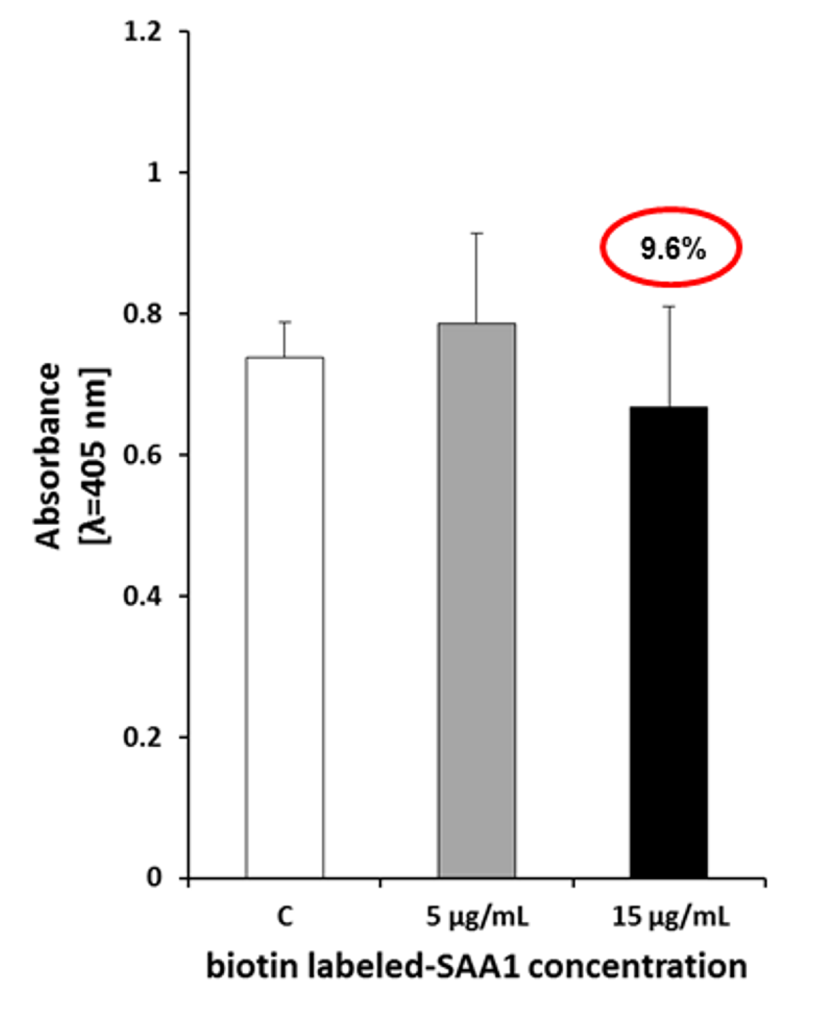


**Figure S4.** Inhibition of biotin-labeled human SAA1 binding by live *Mycobacterium smegmatis* cells in the presence of 1-fold (5 µg/mL) and 3-fold (15 µg/mL) excess of unlabeled homologous protein. C-control bacilli incubated with the addition of culture medium instead of unlabeled SAA1; the percent of inhibition is marked in red circle.

**Table S1.** Primer sequences used for PCR amplification of the gene sequences.

| Amplified region | Primer | Sequence (5’→3’)^a^ | Product size [bp] |
| --- | --- | --- | --- |
| *atpA* sense | *rv1308*/BamHI | cggatccgATGGCTGAGTTGACAATCCCCGC | 1650 |
| *atpA* reverse | *rv1308*/BamHI | cggatccCTATTTCTTCTTCTTCGGCGCCGG |  |
| *abc* sense | *rv2477c*/EcoRI | cgaattcATGGCTGAGTTCATCTACACGATGAAGAAG | 1677 |
| *abc* reverse | *rv2477c*/HindIII | caagcTTAGCCGCGCGTCAGCTTGC |  |
| *tb18.6* sense | *rv2140c*/BamHI | cggatccgATGACAACTTCACCCGACCCG | 531 |
| *tb18.6* reverse | *rv2140c*/HindIII | caagcttCTAACGCTGCTCGTAGGTGCC |  |
| *espB* sense | *rv3881c*/EcoRI | cgaattcATGACGCAGTCGCAGACCGTG | 1383 |
| *espB* reverse | *rv3881c*/HindIII | caagctTCACTTCGACTCCTTACTGTC |  |
| *thiC* sense | *rv0423c*/HindIII | caagcttATGACCATCACCGTTGAACCG | 1644 |
| *thiC* reverse | *rv0423c*/HindIII | caagctTCACTGGGTTATCGGGAGATAC |  |
| *ppiA* sense | *rv0009*/BamHI | cggatccgATGGCAGACTGTGATTCCGTG | 549 |
| *ppiA* reverse | *rv0009*/ HindIII | caagctTCAGGAGATGGTGATCGAC |  |

**^a^** the restriction enzyme recognition sites are underlined

**Table S2.** *Mycobacterium tuberculosis* SAA1 binding protein and peptide list identified by LC/ESI-MS/MS.

| **Fasta Headers** | **Proteins** | **Peptides fa** | **Peptides fb** | **Peptides mtb** | **Mol. Weight [kDa]** | **Sequence Length** | **Sequence Coverage fa [%]** | **Sequence Coverage fb [%]** | **Sequence Coverage mtb [%]** | **Intensity** | **Intensity fa** | **Intensity fb** | **Intensity mtb** |
| --- | --- | --- | --- | --- | --- | --- | --- | --- | --- | --- | --- | --- | --- |
| >Rv0384c_clpB | 1 |  |  | 1 | 92.535 | 848 |  |  | 0.9 | 1.29E+08 |  |  | 1.29E+08 |
| >Rv1308_atpA | 1 | 18 |  | 3 | 59.288 | 549 | 39 |  | 6.2 | 3.60E+08 | 3.35E+08 |  | 2.51E+07 |
| >Rv1310_atpD | 1 |  |  | 1 | 53.094 | 486 |  |  | 2.5 | 3.55E+06 |  |  | 3.55E+06 |
| >Rv1650_pheT | 1 |  |  | 1 | 88.373 | 831 |  |  | 1.2 | 1.04E+05 |  |  | 1.04E+05 |
| >Rv0440_groEL2 | 1 | 13 | 1 |  | 56.726 | 540 | 27.4 | 3,3 |  | 1.18E+08 | 1.18E+08 | 1.50E+05 |  |
| >Rv1133c_metE | 1 | 4 | 1 |  | 81.548 | 759 | 6.6 | 1,3 |  | 5.19E+06 | 5.06E+06 | 1.31E+05 |  |
| >Rv0350_dnaK | 1 | 22 |  |  | 66.83 | 625 | 39 |  |  | 3.69E+08 | 3.69E+08 |  |  |
| >Rv2477c_Rv2477c ABCtransport EttA/ChvD | 1 | 16 |  |  | 61.892 | 558 | 32.6 |  |  | 1.72E+08 | 1.72E+08 |  |  |
| >Rv2455c_Rv2455c | 1 | 15 |  |  | 69.15 | 653 | 31.2 |  |  | 1.48E+08 | 1.48E+08 |  |  |

| **Fasta headers** | **Proteins** | **Peptides** | **Mol. weight [kDa]** | **Sequence length** | **PEP** | **Sequence coverage SAA1 [%]** | **Sequence coverage SAA2 [%]** | **Sequence coverage SAA3 [%]** | **Intensity SAA1** | **Intensity SAA2** | **Intensity SAA3** |
| --- | --- | --- | --- | --- | --- | --- | --- | --- | --- | --- | --- |
| >Rv0440_groEL2 | 1 | 17 | 56.726 | 540 | 5.99E-214 | 44,4 | 20,9 |  | 3,95E+08 | 1,04E+07 |  |
| >Rv3881c_espB | 1 | 10 | 47.593 | 460 | 1.19E-55 |  | 33 |  |  | **2,50E+07** |  |
| >Rv0009_ppiA | 1 | 7 | 19.239 | 182 | 6.15E-94 |  | 12.6 | 54.4 |  | 3,92E+05 | **1,96E+09** |
| >Rv2140c_TB18.6 | 1 | 6 | 18.634 | 176 | 6.21E-54 |  | 9.1 | 63.6 |  | 2,02E+05 | **2,01E+08** |
| >Rv1617_pykA | 1 | 6 | 50.667 | 472 | 3.92E-35 |  | 16.1 |  |  | 9,12E+06 |  |
| >Rv0423c_thiC | 1 | 6 | 59.897 | 547 | 7.93E-85 | 15.2 | 13 |  | **6,36E+06** | 4,27E+06 |  |
| >Rv0957_purH | 1 | 5 | 55.026 | 523 | 2.74E-46 |  | 20.1 |  |  | **3,01E+07** |  |
| >Rv2858c_aldC | 1 | 5 | 48.258 | 455 | 9.60E-30 |  | 11 |  |  | 2,98E+06 |  |
| >Rv3417c_groEL1 | 1 | 5 | 55.877 | 539 | 5.44E-17 | 15.4 | 2.2 |  | 4,37E+06 | 3,23E+05 |  |
| >Rv3841_bfrB | 1 | 4 | 20.442 | 181 | 3.37E-47 |  |  | 28.2 |  |  | 2,77E+07 |
| >Rv3442c_rpsI | 1 | 3 | 16.436 | 151 | 1.21E-05 |  |  | 19.2 |  |  | 4,14E+06 |
| >Rv3418c_groES | 1 | 3 | 10.772 | 100 | 3.05E-05 | 9 | 15 | 32 | 3,60E+05 | 9,30E+05 | 3,83E+06 |
| >Rv2831_echA16 | 1 | 3 | 26.63 | 249 | 6.54E-08 |  |  | 18.5 |  |  | 2,64E+06 |
| >Rv3583c_Rv3583c | 1 | 3 | 17.907 | 162 | 9.21E-15 |  |  | 20.4 |  |  | 2,28E+06 |
| >Rv1829_Rv1829 | 1 | 2 | 18.114 | 164 | 0.000194 |  |  | 14.6 |  |  | 5,71E+06 |
| >Rv0580c_Rv0580c | 1 | 2 | 18.035 | 163 | 1.87E-05 |  |  | 18.4 |  |  | 3,17E+06 |
| >Rv0054_ssb | 1 | 2 | 17.321 | 164 | 1.14E-05 |  |  | 12.8 |  |  | 2,08E+06 |
| >Rv0020c_fhaA | 1 | 2 | 56.88 | 527 | 4.69E-07 |  |  | 4.7 |  |  | 1,32E+06 |
| >Rv1484_inhA | 1 | 2 | 28.528 | 269 | 7.24E-06 | 3,7 | 3.7 | 3 |  | 4,02E+05 | 5,55E+05 |
| >Rv0379_secE2 | 1 | 2 | 7.9661 | 71 | 1.88E-22 |  | 49.3 | 18.3 |  | 9,06E+06 | 2,94E+05 |
| >Rv0685_tuf | 1 | 2 | 43.561 | 396 | 6.32E-28 |  | 8.1 |  |  | 3,35E+06 |  |
| >Rv2213_pepB | 1 | 2 | 53.449 | 515 | 2.17E-08 |  | 4.7 |  |  | 1,85E+06 |  |
| >Rv0753c_mmsA | 1 | 2 | 54.453 | 510 | 1.00E-13 |  | 6.5 |  |  | 1,27E+06 |  |
| >Rv2251_Rv2251 | 1 | 2 | 55.77 | 529 | 2.88E-06 | 8,5 |  |  | **5,62E+06** |  |  |
| >Rv1284_canA | 1 | 1 | 18.157 | 163 | 1.58E-17 |  |  | 15.3 |  |  | 1,45E+06 |

| common background |
| --- |
| identified by => 2 peptides |
| best candidates |
